# Post‐Translational Regulation of CD8^+^ T Cell Fate and Dysfunction in Tumor Immunity

**DOI:** 10.1002/advs.74807

**Published:** 2026-03-12

**Authors:** Zihao Zhou, Xu Chen, Nina Li, Meiyan Zou, Yunfan Lin, Pei Lin, Weiyao Feng, Xinyuan Zhao, Li Cui

**Affiliations:** ^1^ Stomatological Hospital, School of Stomatology Southern Medical University Guangzhou Guangdong China; ^2^ School of Dentistry University of California Los Angeles California USA

**Keywords:** CD8^+^ T cell, metabolic regulation, PTMs, TME, tumor immunotherapy

## Abstract

CD8^+^ T cells are central executors of antitumor immunity, yet their activation, effector differentiation, and long‐term persistence are governed by diverse post‐translational modifications (PTMs). These chemical modifications function as rapid and reversible regulators that link antigenic stimulation, metabolic availability, and inflammatory cues to the transcriptional and chromatin programs that define CD8^+^ T cell fate. Core PTM classes—including phosphorylation, ubiquitination, acetylation, methylation, and glycosylation—precisely tune signaling thresholds, cytotoxic commitment, and memory formation, while emerging metabolism‐responsive modifications such as lactylation directly connect nutrient flux to functional fitness. In solid tumors, chronic antigen exposure, hypoxia, nutrient restriction, and lactate accumulation profoundly remodel these modification networks, stabilizing dysfunction‐associated states, impairing metabolic flexibility, and diminishing cytotoxic capacity. This review integrates current mechanistic understanding of how major PTM pathways coordinate the lifecycle of CD8^+^ T cells—from initial activation to effector acquisition, memory establishment, dysfunction, and exhaustion. We further discuss how the tumor microenvironment reprograms PTM landscapes to reinforce dysfunction and promote immune escape. Finally, we highlight the challenges and future directions in deciphering and targeting PTMs in CD8^+^ T cells. Future efforts to manipulate PTMs hold significant potential to improve cancer immunotherapies by restoring the antitumor efficacy of CD8^+^ T cells within the tumor microenvironment.

## Graphical abstract

This review delineates how post‐translational modifications (PTMs) function as a central regulatory interface governing CD8^+^ T cell activation, differentiation, persistence, and exhaustion in antitumor immunity. By integrating antigenic, metabolic, and microenvironmental cues, diverse PTM programs coordinate transcriptional and chromatin states that define cytotoxic and memory fate decisions. In solid tumors, metabolic stress and chronic stimulation rewire PTM landscapes to constrain functional plasticity and enforce immune dysfunction. The article further highlights emerging PTM‐informed therapeutic strategies to restore durable CD8^+^ T cell–mediated tumor control.

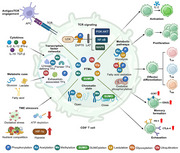

**Abbreviations**
γ_c_
: γ chain
APS: Astragalus polysaccharide
ASXL1: ASXL transcriptional regulator 1
BCL9: BCL9 transcription coactivator
CBX4: Chromobox 4
CCDC134: Coiled‐coil domain containing 134
CMC1: C‐X9‐C motif containing 1
CRL: Cullin‐RING E3 ligase
CUL3: Cullin 3
CUL5: Cullin 5
DHHC9: ZDHHC palmitoyltransferase 9
DUB: Electronic cigarette
FAS: Fatty acid synthase
FBXO38: F‐box protein 38
GCNT1: Glucosaminyl (N‐acetyl) transferase 1
GLI1: GLI family zinc finger 1
GPC3: Glypican 3
GPD1L: Glycerol‐3‐phosphate dehydrogenase 1 like
IBD: Inflammatory bowel disease
ITIM: Immunoreceptor tyrosine‐based inhibitory motif
KAT: Lysine acetyltransferase
KAT2A: Lysine acetyltransferase 2A
KDACs/Sirtuins: Lysine deacetylase
KLF3: Kruppel‐like factor 3
KLHL22: Kelch like family member 22
LCK: Lymphocyte cell‐specific protein‐tyrosine kinase
LXR: Liver X receptor
MARCH5: Membrane associated ring‐CH‐type finger 5
MCT1: Monocarboxylate transporter 1
MDM2: Mouse double minute 2 homolog
Met: Methionine
MGAT5: Alpha‐1,6‐mannosylglycoprotein 6‐beta‐N‐acetylglucosaminyltransferase
NAE1: NEDD8 activating enzyme E1 subunit 1
NEDD8: Neural precursorcell‐expressed developmentally downregulated 8
NFAT: Nuclear factor of activated T cells
Nur77: Nuclear receptor subfamily 4, group A, member 1
OPA1: OPA1 mitochondrial dynamin like GTPase
OX40CD134: CD134
PDCD1: Programmed cell death 1
Peli1: Pellino1
PKCθ: Protein kinase C‐theta
PR‐DUB: Polycomb repressive deubiquitinase
PTBP3: Polypyrimidine region‐ binding protein 3
PTM: Post‐translational modification
RBX1: Ring‐box 1
SAM: S‐adenosylmethionine
SENP1: Sentrin‐specific protease 1
SENP7: SUMO specific peptidase 7
SerpinB9: Serpin family B member 9
SETD2: SET domain containing 2
SHP‐1: Src‐homology 2 domain‐containing protein tyrosine phosphatase 1
SHP‐2: Src‐homology 2 domain‐containing protein tyrosine phosphatase 2
Siglec‐9: Sialic acid‐binding immunoglobulin‐like lectin 9
Sirt3: Sirtuin 3
SLC7A5: Solute carrier family 7 member 5
SLC38A1: Solute carrier family 38 member 1
SP1: Specificity protein 1
SRC1: Steroid receptor coactivator 1
SUMO: Small ubiquitin‐like modifier
TCR: T cell receptor
TIGIT: T cell immune receptor with Ig and ITIM domains
TIM3: T cell immunoglobulin and mucin‐domain containing 3
TME: Tumor microenvironment
TP53: Tumor protein 53
TRAF6: TNF receptor associated factor 6
TRIM21: Tripartite motif containing 21
TSC1: TSC complex subunit 1
TSC2: TSC complex subunit 2
UBC9: Ubiquitin conjugating enzyme 9
UFL1: UFM1‐specific ligase 1
UFM1: Ubiquitin fold modifier 1
USP1: Ubiquitin specific peptidase 1
USP7: Ubiquitin specific peptidase 7
USP24: Ubiquitin specific peptidase 24
VCP: Valosin‐containing protein
VAV1: Vav guanine nucleotide exchange factor 1
YAP1: Yes1 associated transcriptional regulator
YME1L1: YME1 like 1 ATPase
ZAP70: Zeta chain of T cell receptor associated protein kinase 70

## 1 Introduction

CD8^+^ T cells represent the most potent cytotoxic arm of the adaptive immune system and are essential for antiviral defense, tumor control, and allogeneic graft rejection [1]. From the moment they encounter antigen in secondary lymphoid organs, CD8^+^ T cells initiate a precisely coordinated sequence of transitions that include activation, proliferation, effector differentiation, and the establishment of long‐lived memory pools [2]. These fate decisions arise from the integration of transcriptional, epigenetic, metabolic, and signaling programs, which together ensure that T cells respond rapidly yet appropriately to fluctuating immunological demands [3, 4].

At a conceptual level, CD8^+^ T cell fate is governed by the duration and intensity of antigen exposure, which instructs distinct differentiation trajectories through coordinated transcriptional, metabolic, and epigenetic programs. During acute infection, naïve CD8^+^ T cells undergo clonal expansion and differentiate into short‐lived effector cells and memory precursor populations. These fate decisions are shaped by inflammatory cues, TCR signal strength, and metabolic state, which converge on lineage‐defining transcription factors to balance terminal effector function with long‐term persistence [5]. Effector subsets mediate pathogen clearance, whereas memory cells retain self‐renewal capacity and multipotency, reflecting a plastic differentiation program supported by dynamic chromatin remodeling.

In contrast, persistent antigen stimulation, as observed in chronic viral infection, drives CD8^+^ T cells into an exhaustion differentiation program characterized by sustained inhibitory receptor expression, progressive attenuation of effector function, hierarchical developmental progression, and stable transcriptional and epigenetic remodeling [6]. TCF1^+^ progenitor populations sustain the exhausted compartment and give rise to intermediate and terminal subsets with progressively stabilized chromatin landscapes and reduced proliferative capacity [7]. In solid tumors, CD8^+^ T cells frequently acquire a dysfunctional state that shares core features of exhaustion but is additionally shaped by tumor‐specific microenvironmental pressures, including metabolic deprivation, hypoxia, and suppressive cytokine signaling. Tumor‐associated dysfunction therefore, reflects an exhaustion‐related differentiation state modified by local environmental constraints rather than a state entirely equivalent to virally induced exhaustion. Notably, tumor‐infiltrating CD8^+^ T cells can occupy progenitor‐like plastic chromatin states responsive to checkpoint blockade or more fixed states resistant to reprogramming, underscoring the influence of microenvironmental factors on lineage stability and therapeutic reversibility [8]. Collectively, classical exhaustion in chronic infection and tumor‐associated dysfunction arises from overlapping differentiation programs but are distinguished by the contextual signals that shape their transcriptional, metabolic, and epigenetic landscapes [9].

**FIGURE 1:**
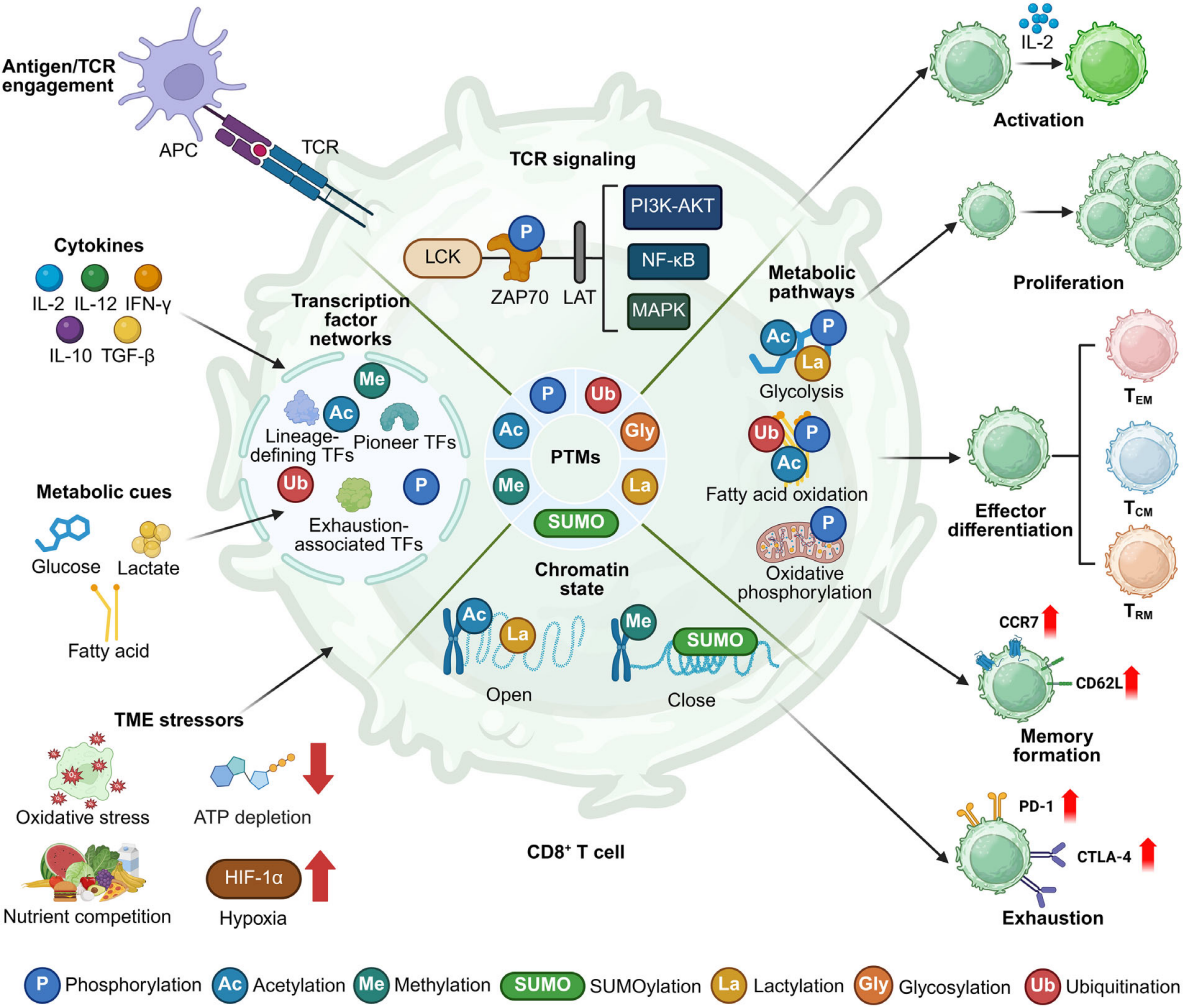
PTMs orchestrate signal integration and fate decisions in CD8^+^ T cells. Antigen recognition by the T cell receptor (TCR) initiates proximal signaling cascades involving LCK and ZAP70, which propagate through PI3K–AKT, NF‐κB, and MAPK pathways to drive CD8^+^ T cell activation. These signals are dynamically modulated by PTMs, including phosphorylation, acetylation, methylation, ubiquitination, SUMOylation, lactylation, and glycosylation, which collectively regulate protein activity, stability, and interaction networks. In parallel, cytokine cues and metabolic inputs—such as glucose, fatty acids, and lactate—converge on transcription factor networks and chromatin states through PTM‐dependent mechanisms, shaping lineage‐defining, pioneer, and exhaustion‐associated transcriptional programs. PTMs further couple metabolic pathway engagement, including glycolysis, fatty acid oxidation, and oxidative phosphorylation, to effector differentiation, proliferation, and memory formation. Within the tumor microenvironment, chronic antigen stimulation, hypoxia, nutrient limitation, oxidative stress, and immunosuppressive cytokines perturb PTM homeostasis, progressively biasing CD8^+^ T cells toward dysfunctional and exhausted states characterized by sustained inhibitory receptor expression. Together, PTMs function as a central integrative layer that encodes signaling, metabolic, and environmental information into coordinated transcriptional, epigenetic, and functional outcomes, thereby governing the balance between effector function, memory formation, and exhaustion in CD8^+^ T cells. Created in https://BioRender.com.

Within this multilayered regulatory system, post‐translational modifications (PTMs) form a uniquely dynamic and plastic control axis. By rapidly altering protein activity, stability, and interaction networks, PTMs enable CD8^+^ T cells to remodel their functional state in real time, aligning cellular behavior with the biochemical and immunological context (Figure 1) [10, 11].

A growing body of evidence highlights the centrality of PTMs in coordinating key aspects of CD8^+^ T cell biology. Classical modifications such as phosphorylation govern the amplitude and duration of T cell receptor (TCR) signaling by modulating the activity of lymphocyte cell‐specific protein‐tyrosine kinase (Lck), zeta chain of t cell receptor associated protein kinase 70 (ZAP70), and downstream PI3K–AKT–mTOR, NF‐κB and MAPK pathways [12, 13]. Ubiquitination and deubiquitination regulate the turnover of signaling intermediates, transcription factors, and metabolic enzymes through diverse chain architectures built on multiple lysine residues [14, 15]. These canonical PTMs establish the foundational circuitry that dictates activation thresholds, clonal expansion, and effector acquisition. In parallel, an expanding class of “non‐classical” and metabolism‐sensitive PTMs—such as lactylation, glycosylation, and SUMOylation—links nutrient availability, lactate accumulation, and redox state to transcriptional and chromatin remodeling programs [16]. Through these mechanisms, PTMs orchestrate the metabolic flexibility, effector gene expression, and epigenetic plasticity that collectively define T cell functional states.

The tumor microenvironment (TME) imposes profound disturbances on these regulatory layers. CD8^+^ T cells infiltrating tumors are exposed to chronic antigen stimulation, hypoxia, nutrient scarcity, high lactate concentrations, elevated free fatty acids, and a milieu of immunosuppressive cytokines. These pressures reshape the PTM landscape at multiple levels. Hypoxia‐driven alterations in prolyl hydroxylase activity influence metabolic preference and proliferative capacity [17]. Excess lactate enhances histone and non‐histone lactylation, promoting dysfunction‐associated transcriptional modules [18, 19]. Depletion of acetyl‐CoA and S‐adenosylmethionine (SAM) restricts acetylation and methylation, altering chromatin accessibility and gene‐expression programs [20]. Oxidative stress disrupts the balance between ubiquitination and deubiquitination, destabilizing signaling components and attenuating TCR responsiveness [21]. Collectively, these perturbations convert PTMs into molecular encoders of environmental stress, driving the establishment and stabilization of dysfunctional and exhausted CD8^+^ T cell states.

These insights have converged on a unifying framework: CD8^+^ T cell fate is fundamentally a multidimensional signal‐integration process, and PTMs sit at the core of this architecture. They maintain developmental and functional balance under physiological conditions, yet under pathological stress—particularly within tumors—they encode persistent metabolic and inflammatory cues into durable transcriptional and epigenetic reprogramming. Understanding the logic of PTM‐mediated regulation is therefore essential for deciphering how CD8^+^ T cells acquire, sustain, or lose effector functionality. Building on this conceptual foundation, this review offers an integrative framework that unifies PTM‐dependent regulation of CD8^+^ T cell activation, metabolic wiring, effector programming, memory formation, and exhaustion—processes that have seldom been examined within a single coherent context. We further delineate how the TME imposes selective metabolic and inflammatory pressures that reshape these modification networks and progressively confine CD8^+^ T cells to dysfunctional states. By linking these mechanistic insights to emerging therapeutic strategies that modulate PTM‐regulated pathways, this review provides a consolidated perspective that underscores PTMs as an overlooked yet essential regulatory dimension in the control of antitumor CD8^+^ T cell immunity.

## 2 Overview of PTM

**FIGURE 2:**
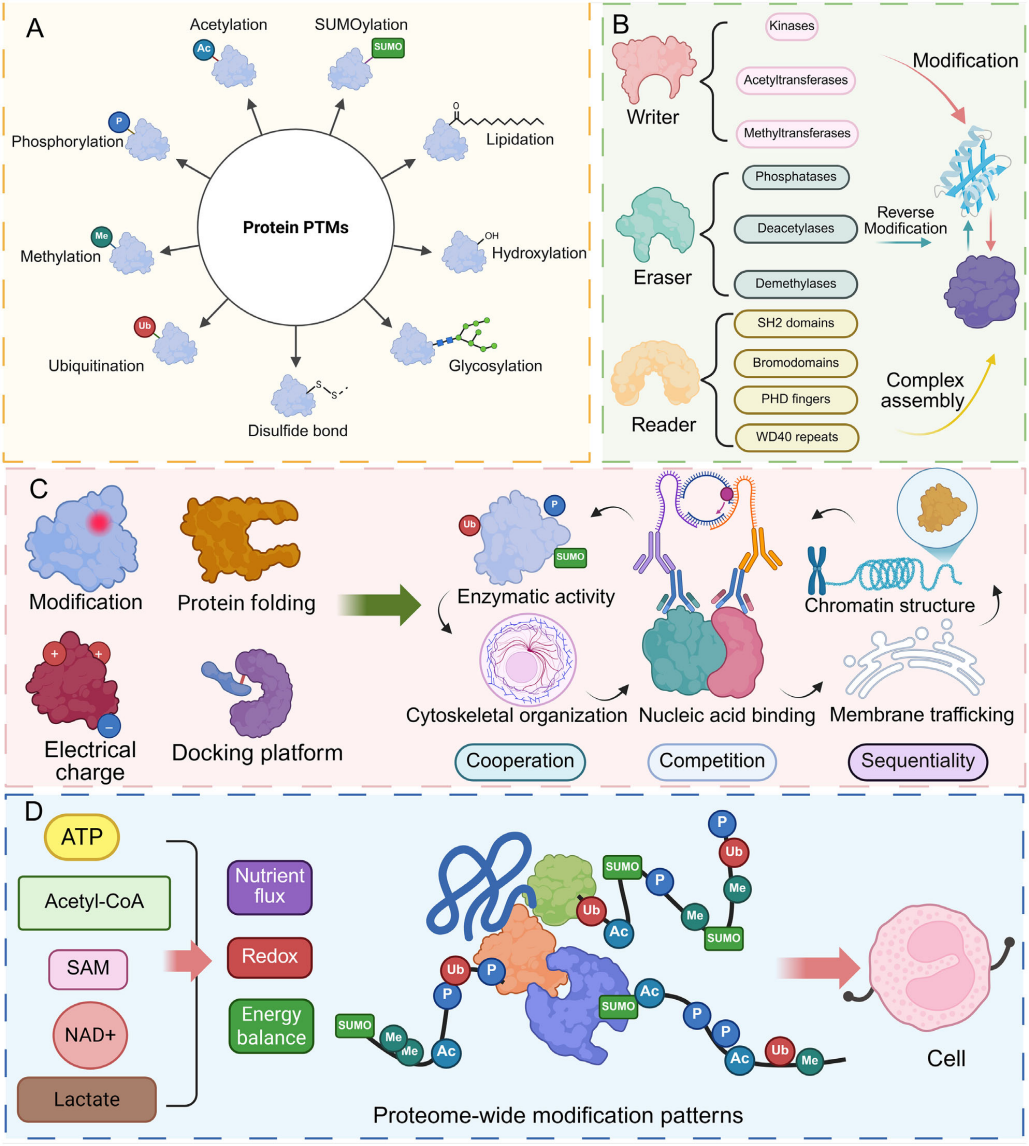
Core regulatory networks, functional mechanisms, and metabolic integration of protein PTMs. (A) Schematic overview of representative types of protein post‐translational modifications, including phosphorylation, acetylation, methylation, ubiquitination, SUMOylation, glycosylation, lipidation, hydroxylation, and disulfide bond formation. (B) Dynamic regulation of PTMs is orchestrated by a modular “writer–eraser–reader” enzyme network. Writer enzymes catalyze the installation of specific modifications, eraser enzymes mediate their removal or reversal, and reader domains selectively recognize defined modification states, thereby recruiting downstream effectors or facilitating higher‐order protein complex assembly. (C) Functional regulatory principles of PTMs. By modifying catalytic residues, altering electrostatic surface properties, reshaping local protein conformation, or creating molecular docking platforms, PTMs establish a multilayered regulatory system that broadly influences protein function and cellular processes, including enzymatic activity, nucleic acid binding, chromatin organization, cytoskeletal dynamics, and membrane trafficking. Combinatorial logic among multiple modifications—through cooperative, competitive, or sequential interactions—enables a limited set of PTMs to generate highly precise and context‐dependent biological outcomes. (D) Metabolic integration of PTMs. The availability of metabolic cofactors and intermediates, such as ATP, acetyl‐CoA, S‐adenosylmethionine (SAM), NAD^+^, and lactate‐associated acyl‐CoA species, directly couples nutrient flux, redox status, and energy balance to proteome‐wide PTM patterns, thereby coordinating appropriate cellular responses under physiological and pathological conditions. Created in https://BioRender.com.

PTMs are covalent chemical alterations introduced to proteins after translation, profoundly expanding the regulatory and structural complexity of the proteome [22]. Although the human genome encodes only ∼20 000 genes, PTMs generate more than one million distinct proteoforms, enabling proteins to adopt diverse conformations, activities, and interaction profiles [23, 24]. This functional expansion arises from a rapidly growing repertoire of over 500 PTM types identified to date, spanning small‐group additions—such as phosphorylation, acetylation, methylation, hydroxylation, and O‐GlcNAcylation—to larger conjugates including ubiquitin, small ubiquitin‐like modifiers (SUMO), and other ubiquitin‐like modifiers [25]. The continual discovery of novel modifications, including lactylation, succinylation, and β‐hydroxybutyrylation, highlights the proteome's remarkable chemical adaptability and the extensive regulatory potential embedded within PTM systems (Figure 2A) [26].

The installation, removal, and interpretation of PTMs are controlled by expansive enzyme networks that confer substrate specificity, spatial–temporal precision, and reversibility (Figure 2B). Writer enzymes, such as kinases, acetyltransferases, and methyltransferases, introduce modifications, while erasers—including phosphatases, deacetylases, and demethylases—reverse them in a context‐dependent manner [27, 28]. Protein‐conjugation PTMs, such as ubiquitination, rely on hierarchical cascades involving E1 activation enzymes, E2 conjugases, and a diverse family of E3 ligases that determine target selectivity [29]. In parallel, reader domains—including SH2, bromodomains, chromodomains, PHD fingers, and WD40 repeats—decode modification states to recruit effector proteins or assemble higher‐order complexes [30]. This modular enzymatic architecture ensures that PTMs can be rapidly reconfigured in response to developmental cues, stress signals, metabolic fluctuations, or extracellular stimuli.

Functionally, PTMs constitute a multilayered regulatory system that influences nearly every aspect of protein and cellular physiology (Figure 2C). By modifying catalytic sites, altering surface charge, reshaping local protein folds, or creating docking platforms, PTMs modulate enzymatic activity, protein stability, nucleic acid binding, chromatin structure, cytoskeletal organization, and membrane trafficking [31, 32, 33]. Many proteins harbor multiple modifications simultaneously, and the interactions among them produce an integrated “PTM code”. Distinct PTMs may act cooperatively to amplify signaling, competitively to restrict access to regulatory sites, or sequentially to guide ordered progression of biochemical events [34, 35, 36]. This combinatorial logic allows PTMs to generate highly precise and context‐specific outcomes despite the finite number of modification types, enabling dynamic tuning of signaling strength, transcriptional programs, and stress‐adaptation responses.

A defining property of PTMs is their deep integration with cellular metabolism, positioning them as sensitive molecular interpreters of the biochemical environment (Figure 2D). The availability of metabolic donors—such as ATP for phosphorylation, acetyl‐CoA for acetylation, SAM for methylation, NAD^+^ for ADP‐ribosylation, and lactate for lactylation—directly links nutrient flux, redox state, and energy balance to proteome‐wide modification patterns [37, 38, 39]. Metabolite levels regulate not only the rate of PTM installation but also the localization and activity of enzymes. This metabolic coupling enables PTMs to convert environmental perturbations into coordinated shifts in chromatin accessibility, transcription factor activity, organelle communication, and protein turnover [40, 41]. Through these integrated mechanisms, PTMs form a dynamic and adaptive regulatory framework that maintains proteome plasticity and ensures appropriate cellular responses across diverse physiological and pathological contexts.

## 3 PTMs Regulating CD8^+^ T Cell Function in Cancer

### 3.1 Ubiquitination

#### 3.1.1 Ubiquitination‐Mediated Regulation of CD8^+^ T Cell Function in Cancer

E3 ubiquitin ligases constitute a multilayered regulatory network that calibrates receptor turnover, signal amplitude, and metabolic competence in CD8^+^ T cells, thereby determining the balance between activation, dysfunction, and long‐term persistence (Table 1) [42, 43]. Among them, the RING‐type ligase Cbl‐b functions as a pivotal molecular brake on CD8^+^ T cell effector activity within tumors.

**TABLE 1:** PTM‐ and metabolite‐mediated control of CD8^+^ T cell physiology and antitumor immunity.

PTM category	Regulatory molecule(s)	Molecular mechanism	Effect on CD8^+^ T cell function	Functional / physiological outcome	Refs.
Ubiquitination	CCDC134	Prevents CD3ε ubiquitin‐mediated degradation, preserves proximal TCR signaling	TCR signal initiation, antigen‐induced activation, proliferative response	Reduced TCR signal strength, impaired activation, and antitumor responses	[44]
	Cbl‐b	Ubiquitinates TCR/CD226 signaling components (via CD226, Y319)	Drives dysfunction (PD‐1^+^TIM‐3^+^), suppresses cytokines and cytotoxicity	Promotes immune evasion, Cbl‐b loss or inhibition enhances CAR‐T persistence and tumor control	[45, 46, 47]
	MDM2	Competes with c‐Cbl to stabilize STAT5	Maintains survival and IFN‐γ production	Enhances antitumor immunity, synergizes with checkpoint blockade	[48]
	TRIM21	K63‐linked ubiquitination stabilizes PD‐1	Suppresses cytotoxic activation, enforces dysfunction	Facilitates immune evasion, TRIM21 loss enhances checkpoint and CAR‐T efficacy	[51]
	KLHL22	K48‐linked ubiquitination of immature PD‐1	Limits PD‐1 accumulation, preserves effector function	Restrains tumor progression, KLHL22 loss promotes immune suppression	[52]
	PTBP3–ΔIL‐18–FBXO38	Mediates PD‐1 degradation, suppressed by the ΔIL‐18	Sustains PD‐1 inhibitory signaling, impairs cytotoxicity	Promotes immune evasion and tumor progression	[53]
	PD‐1–BATF–MARCH5, SHP‐2	K27‐linked ubiquitination and dephosphorylation of γ_c_	Attenuates IL‐2 signaling and effector activation	Limits antitumor immunity, PD‐1 or MARCH5 blockade restores cytokine responsiveness	[54]
	TRAF6	K63‐linked ubiquitination and lysosomal degradation of CTLA‐4	Relieves inhibition, enhances effector function	Strengthens antitumor immunity, improves checkpoint therapy efficacy	[55]
	Peli1	K48‐linked ubiquitination and degradation of PKCθ	Suppresses effector activity, promotes dysfunction	Peli1 loss enhances cytokine production and antitumor CD8^+^ T cell function	[56]
	β‐TrCP	Ubiquitination and degradation of YAP1	Impairs amino acid uptake and mTOR signaling, drives dysfunction	Restoring β‐TrCP function enhances metabolism and antitumor immunity	[57]
	Peli1	Non‐degradative ubiquitination of TSC1, stabilizes TSC1–TSC2 and suppresses mTORC1	Limits glycolysis and metabolic fitness	Peli1 loss enhances T cell metabolism, infiltration, and antitumor activity	[58]
Deubiquitination	USP1	Stabilizes Id2/Id3, prevents proteasomal turnover	Lineage maintenance, memory precursor stability, recall expansion	Compromised memory maintenance, weakened recall cytotoxicity	[61]
	USP30	Removes Parkin‐dependent ubiquitin marks on transferred mitochondria, prevents mitophagy in CD8^+^ T cells	Mitochondrial quality control and metabolic programming in CD8^+^ T cells	CD8^+^ T cell senescence with impaired effector and memory function and reduced immunotherapy responsiveness	[62]
	USP24	K48‐linked deubiquitination stabilizes PD‐1	Sustains dysfunction, suppresses cytotoxicity	USP24 inhibition restores effector function and enhances checkpoint therapy, high USP24 correlates with immune dysfunction	[63]
	USP7	Deubiquitinates CMC1, stabilizing mitochondrial complex IV	Promotes terminal differentiation and dysfunction	CMC1 loss preserves memory‐like CD8^+^ T cells, USP7–CMC1 axis limits antitumor immunity	[64]
	ASXL1	Modulates H2AK119 ubiquitination to preserve chromatin accessibility	Maintains progenitor‐exhausted CD8^+^ T cells	ASXL1 loss enhances persistence and synergizes with PD‐L1 blockade for durable tumor control	[65]
NEDDylation	NAE1, SENP8	Modifies glycolytic enzymes and sustains proteomic/metabolic programs via NFATc1	Supports activation, proliferation, effector differentiation, and metabolic fitness	Loss impairs antitumor function, SENP8 inhibition or NAE1 overexpression enhances CAR‐T cytotoxicity and tumor clearance	[68, 69]
	NAE inhibition (pevonedistat)	Suppresses CRL activity, stabilizes HIF‐1α, amplifies interferon/chemokine signaling	Promotes proinflammatory, cytotoxic phenotype, increases TNFα and IFNγ	Enhances tumor infiltration and immune activation, delays tumor progression, synergizes with PD‐1 blockade	[70]
	CUL5–PCMTD2	Neddylation‐activated E3 ligase restrains TCR and IL‐2 signaling	Limits cytokine responsiveness and effector differentiation	CUL5 loss or neddylation blockade enhances antitumor activity, CTLA‐4 inactivation further boosts efficacy	[71]
UFMylation	UFL1	UFMylates PD‐1 to prevent ubiquitin‐mediated degradation, Thr536 phosphorylation disrupts interaction	Maintains PD‐1 stability, suppresses activation	UFL1 loss enhances cytotoxic infiltration and antitumor immunity, sensitizes tumors to CTLA‐4 blockade	[72]
	TP53, UFM1, exosomal SerpinB9	Exosomal SerpinB9 blocks TP53 UFMylation, reducing granzyme B	Suppresses cytotoxic activation	Microbiota‐lipid‐UFMylation axis limits CD8^+^ T cell antitumor function in multiple myeloma	[73]
SUMOylation	UBC9	Maintains IL‐7 survival signaling, supports NFAT nuclear retention	Thymocyte maturation, CD4^+^/CD8^+^ single‐positive populations development	Defective thymocyte maturation and disrupted CD8^+^ T cell homeostasis	[77]
	RORγt	SUMOylation at lysine 31 recruits KAT2A–SRC1 coactivator complex, enhancing transcriptional activity	Lineage programming, Th17 differentiation, thymic CD8^+^ immature single‐positive cells maturation	Impaired lineage programming and altered lymphoid development	[78]
	CBX4	SUMOylates SP1/KLF3 to elevate Aldolase B, suppress Akt phosphorylation and glycolysis	Restrains metabolic fitness and effector function	CBX4 loss restores glycolysis and enhances PD‐1 blockade responsiveness	[79]
	LXR	Cholesterol‐induced SUMOylation of LXR reduces p65 recruitment to Il9 promoter	Inhibits Tc9 cell differentiation and function	Limits antitumor immunity	[80]
	SUMO‐activating enzyme (inhibited by TAK‐981)	Blocks SUMO conjugation, affecting transcription, DNA repair, and signaling	Preserves TCR activation, enhances IFN‐γ secretion	Reduces Treg differentiation, reprograms T cell responses to boost antitumor immunity	[81]
DeSUMOylation	SENP7	DeSUMOylates PTEN in response to oxidative stress, preventing metabolic suppression	Maintains glycolysis, OXPHOS, proliferation, and effector function	SENP7 loss reduces metabolic fitness and antitumor activity	[82]
	SENP1, Sirt3	Increases Sirt3 deacetylase activity, reduces YME1L1 acetylation, limits OPA1 cleavage, promotes mitochondrial fusion and OXPHOS	Metabolic remodeling, memory precursor formation, mitochondrial homeostasis	Improved mitochondrial integrity and strengthened memory formation	[83]
Glycosylation	GCNT1	Enhances core‐2 O‐glycan formation on CD43, modulates Notch‐dependent signaling	CD8^+^ T cell activation, cytokine induction, effector differentiation	Enhanced cytokine production and effector potential	[84]
	MGAT5	β1,6‐GlcNAc branching of surface proteins	Promotes exhaustion (PD‐1^+^TIM‐3^+^) and limits infiltration	MGAT5 loss restores effector function and tumor killing, enhances CAR‐T efficacy, drives colitis‐associated tumor progression, elevated glycosylation predicts cancer risk	[85, 86]
	Oligosaccharyltransferase complex	N‐glycan transfer supports IFN‐γ–driven effector programs	Loss impairs cytokine production and accelerates dysfunction	Restoration revives cytotoxicity and curbs tumor progression, highlights context‐dependent effects	[87]
	Rab37	Directs PD‐1 vesicular trafficking to plasma membrane, dependent on PD‐1 glycosylation	Maintains dysfunction, suppresses proliferation and cytotoxicity	Rab37 loss restores effector function, high Rab37–PD‐1–TIM3 correlates with advanced disease and poor survival	[88]
	Fut8	Core fucosylation stabilizes PD‐1 by preventing ubiquitination	Loss reduces PD‐1, enhances activation and cytotoxicity	Fut8 deficiency boosts tumor clearance and antitumor immunity	[89]
	GSK3β	N‐glycosylation–dependent blockade of GSK3β–β‐TrCP–mediated PD‐L1 ubiquitination	Sustained PD‐1 signaling suppresses CD8^+^ T cell activation and cytotoxicity	Enhances tumor immune escape and reduces responsiveness to PD‐1 blockade	[90]
Phosphorylation	CD226, CD28 (inhibited by PD‐1/TIGIT)	TIM‐dependent PD‐1 inhibition of CD226/CD28 phosphorylation, TIGIT blocks CD226–CD155 engagement	Restrains effector expansion and activation	Dual PD‐1/TIGIT blockade restores CD226 signaling and enhances antitumor CD8^+^ T cell function	[93]
	B7‐H4	Inhibits AKT and eNOS phosphorylation	Induces early T cell dysfunction	Limits antitumor immunity and correlates with reduced T cell density	[94]
	BCL9	Inhibition increases VAV1 phosphorylation, modulates GLI1/PATCH to upregulate tumor CD155	Enhances CD8^+^ T cell infiltration and activation	BCL9 suppression improves anti‐PD‐1 response, high BCL9 correlates with reduced CD226 and poor outcomes	[95]
	VCP	GPD1L‐mediated accumulation of glycerol‐3‐phosphate promotes LCK Tyr505 phosphorylation	Inhibits TCR signaling, suppresses activation and cytotoxicity	VCP targeting restores CD8^+^ T cell function and enhances anti‐PD‐1 efficacy	[96]
	Siglec‐9	Glycan‐dependent engagement triggers SHP‐1 phosphorylation	Suppresses TCR signaling, cytokine release, and cytotoxicity	Limits activation of tumor‐resident CD8^+^ T cells and dampens antitumor immunity	[97]
	STAT3 (activated by IL‐21 or synthetic IL‐21R)	Cytokine‐mediated phosphorylation promotes proliferation and memory differentiation, reduces PD‐1 and apoptosis	Enhances persistence and cytotoxicity of TCR‐T cells	Improves antitumor efficacy, especially under repeated antigen stimulation	[98]
	mTOR (activated by butyrate)	Enhances mTOR phosphorylation, TCR expression, and IFN‐γ production	Promotes dual effector–memory phenotype, sustains cytotoxicity	Butyrate‐conditioned CD8^+^ T cells accumulate in tumors and improve tumor control	[99]
	LCK	Metabolite‐enhanced LCK phosphorylation (Tyr394/Tyr505) strengthens TCR signaling	Increases effector cytokine production, cytotoxicity, and activation	Enhances antitumor immunity, oral supplementation or metabolite availability suppresses tumor growth	[100, 101]
	STAT5 (activated by APS)	Promotes STAT5 phosphorylation	Enhances CAR‐T proliferation, migration, memory differentiation, and effector function	Boosts persistence and antitumor activity of GPC3‐targeted CAR‐T cells, upregulates intratumoral CXCL9/10	[102]
	ZAP70 (activated by tetracyclines)	Enhances ZAP70 phosphorylation and early TCR signaling	Increases CD69/Nur77 expression, granzyme B, and IFN‐γ, strengthens cytotoxicity	Enhances antitumor CD8^+^ T cell immunity independent of PD‐1 blockade	[103]
Palmitoylation	Riplet, STAT3	Riplet loss stabilizes FASN, palmitic acid promotes STAT3 palmitoylation	Drives exhaustion and terminal dysfunction	Riplet deficiency induces metabolic‐driven T cell dysfunction and resistance to anti‐PD‐1, FASN inhibition restores antitumor immunity	[106]
	DHHC9	Palmitoylates TIM‐3 at Cys^296^, preventing HRD1‐mediated ubiquitination	Maintains TIM‐3 stability and induces T cell dysfunction	Disruption restores CAR‐T/NK activity, high DHHC9/TIM‐3 correlates with poor prognosis	[107]
Lactylation	H3K18la	Lactate‐induced H3K18 lactylation induces Nur77, sustaining TCR signaling	Impairs antigen recognition and cytotoxicity	Suppression of lactate restores CD8^+^ T cell function and enhances PD‐1 blockade efficacy	[110]
	H3 lactylation (via MCT1‐mediated lactate transport)	Glycolysis‐driven lactate induces histone lysine lactylation, elevating PD‐1	Alters activation dynamics, modulates dysfunction	Sensitizes CD8^+^ T cells to checkpoint blockade, enhances anti–PD‐1 efficacy in obesity and tumor models	[111]
Acetylation	SIRT2	Deacetylates metabolic regulators (GSK3β) to enhance oxidative metabolism	Promotes effector memory differentiation and metabolic fitness	Loss reduces effector memory, weakens CD8^+^ T cell function, and impairs antitumor immunity	[113]
	Vitamin D receptor (1α,25(OH)_2_D_3_)	Promotes H3K27 acetylation at CD28 and CpG methylation of PDCD1, represses PD‐1, TIM‐3, TIGIT transcription	Enhances cytotoxicity and Th1 cytokine production	Restores CD8^+^ T cell function and reverses dysfunction‐associated signaling	[114]
Methylation	KCa3.1 channel	Early Met‐dependent arginine methylation modulates calcium flux and transcription	Determines exhaustion and effector fate, supports long‐term functional imprinting	Couples nutrient sensing to antitumor potential, offers metabolic intervention opportunities	[118]
	DNMT3A	DNA methylation–mediated repression of stemness programs	Reduces proliferation, persistence, and effector function under chronic antigen exposure	Impaires CAR‐T cell durability and antitumor efficacy	[119]

In parallel to inhibitory ubiquitin ligases that dampen effector signaling, effective antitumor immunity also relies on ubiquitin‐regulated mechanisms that preserve proximal TCR signal integrity. For instance, loss of coiled‐coil domain containing 134 (CCDC134) impairs peripheral T cell homeostasis and attenuates TCR‐induced activation and proliferation. Mechanistically, CCDC134 associates with CD3ε and prevents its ubiquitin‐mediated degradation, thereby preserving proximal TCR signaling. CCDC134‐deficient T cells exhibit diminished responses to antigenic stimulation and fail to mount effective inflammatory or antitumor immunity, highlighting a critical role for CCDC134 in regulating TCR signal strength and maintaining functional CD8^+^ T cell responses [44].

**FIGURE 3:**
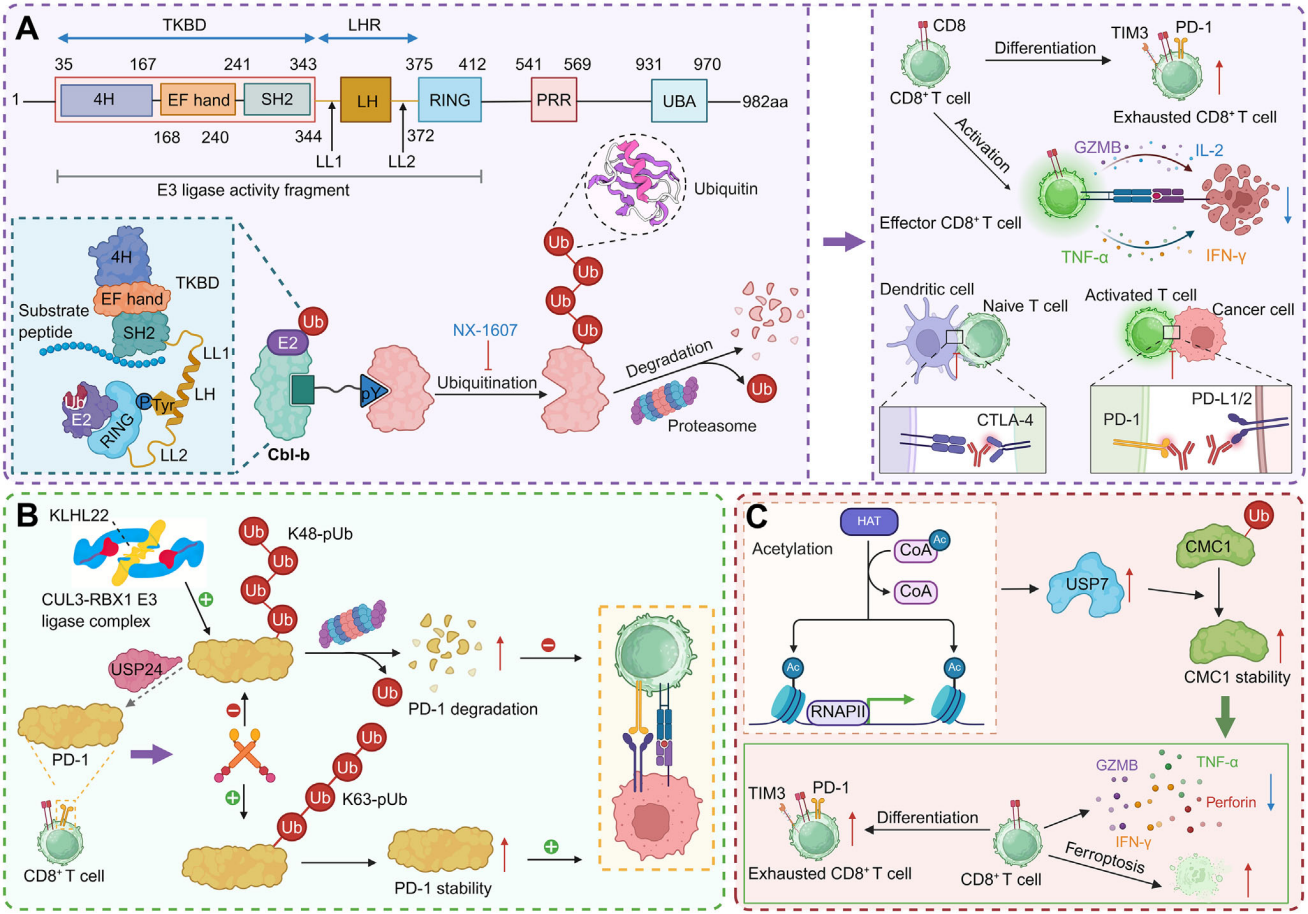
Ubiquitin‐dependent regulation of CD8^+^ T cell dysfunction and effector function in cancer. (A) The E3 ubiquitin ligase Cbl‐b acts as a dominant negative regulator of CD8^+^ T cell activation by ubiquitinating core signaling receptors, including CD226, thereby limiting TCR signaling, effector differentiation, and cytokine production. Genetic deletion or pharmacological inhibition of Cbl‐b (e.g., NX‐1607) restores MAPK pathway activity and enhances IFN‐γ and TNF‐α secretion, promoting antitumor immunity. (B) PD‐1 stability is tightly controlled by antagonistic ubiquitin pathways. TRIM21 mediates K63‐linked ubiquitination to maintain PD‐1 expression, whereas the KLHL22‐CUL3‐RBX1 E3 ligase complex promotes K48‐linked ubiquitination and proteasomal degradation of immature PD‐1. In parallel, the deubiquitinase USP24 counteracts degradative ubiquitination, sustaining PD‐1 abundance and inhibitory signaling in exhausted CD8^+^ T cells. (C) Ubiquitin‐dependent control of metabolic and mitochondrial programs further enforces CD8^+^ T cell dysfunction in the tumor microenvironment. The deubiquitinase USP7 stabilizes mitochondrial regulators such as CMC1, promoting metabolic stress, terminal differentiation, and exhaustion, thereby constraining long‐term effector persistence and antitumor function. Created in https://BioRender.com.

Cbl‐b upregulation in exhausted T cells promotes PD‐1^+^TIM‐3^+^ (T cell immunoglobulin and mucin‐domain containing‐3) phenotypes and cytokine suppression through ubiquitin‐dependent signaling. Genetic deletion of Cbl‐b enhances effector cytokine production, reduces dysfunction markers, and enhances cytotoxicity. In CAR‐T cells, Cbl‐b loss confers resistance to functional decline, sustaining interferon‐γ and TNF‐α expression and improving tumor clearance (Figure 3A) [45]. Similarly, pharmacologic inhibition of Cbl‐b by NX‐1607 relieves its suppressive control over T cell signaling, amplifying PLCγ1 and ERK1/2 phosphorylation to sustain MAPK‐driven activation and cytokine production. This increased signaling enhances CD69 expression and effector differentiation, leading to robust T cell infiltration and tumor suppression in vivo [46]. Notably, tumor‐expressed CD155 induces phosphorylation of CD226 at tyrosine 319 by Src kinases, enabling Cbl‐b–mediated ubiquitination and proteasomal degradation of CD226 in CD8^+^ T cells. Loss of surface CD226 diminishes cytotoxic function and fosters resistance to immune checkpoint blockade, whereas preventing Y319 phosphorylation preserves receptor stability and enhances antitumor activity [47]. In parallel, mouse double minute 2 homolog (MDM2) preserves STAT5 stability in tumor‐infiltrating CD8^+^ T cells by competing with c‐Cbl and preventing STAT5 degradation, thereby sustaining survival and effector function. Pharmacologic activation of MDM2 with APG‐115 enhances STAT5 signaling, boosts interferon‐γ production, and synergizes with checkpoint blockade, effects dependent on p53–MDM2 signaling within T cells [48].

Beyond mechanistic and pharmacologic insights, extensive genetic and in vivo evidence further establishes Cbl‐b as a central downstream integrator of immune checkpoint signaling in T cells. Consistent with its role as a T cell–intrinsic inhibitory ligase, Cbl‐b operates downstream of the PD‐1/PD‐L1 signaling pathway, with Cbl‐b deficiency in T cells conferring resistance to PD‐L1–mediated inhibition. Cbl‐b‐deficient (Cbl‐b^−/−^) T cells show enhanced proliferation and IFN‐γ production in response to tumor antigens. In vivo, Cbl‐b^−/−^ mice develop significantly fewer liver metastases in a B16 melanoma model without anti‐PD‐1 treatment, suggesting that Cbl‐b modulates the PD‐1/PD‐L1 checkpoint to impact immune evasion. Cbl‐b also negatively regulates T cell responses in multiple contexts. It has been established as a key negative regulator of T cell‐dependent tumor immunity, with Cbl‐b^−/−^ T cells exhibiting co‐stimulation independence, hyper‐reactivity, and resistance to PD‐L1/PD‐1 and TGF‐β signaling. Cbl‐b ablation in CD8^+^ T cells promotes robust antitumor responses, even without CD28 co‐stimulation, and enhances spontaneous tumor rejection in murine models [49]. Additionally, Cbl‐b^−/−^ mice efficiently reject both transplanted and spontaneous tumors, and therapeutic transfer of naive Cbl‐b^−/−^ CD8^+^ T cells results in complete tumor eradication and long‐lasting anticancer memory. These findings position Cbl‐b as a critical modulator of immune checkpoints, including the PD‐1/PD‐L1 pathway, suggesting that targeting Cbl‐b could enhance antitumor immunity by bypassing multiple immune regulatory mechanisms, including PD‐L1, TGF‐β, and regulatory T cells [50].

Other E3 ligases exert highly selective control over immune checkpoints, most notably PD‐1, which itself represents a ubiquitin‐sensitive hub that governs activation thresholds and dysfunction trajectories. For instance, tripartite motif containing 21 (TRIM21) enforces PD‐1 stability in CD8^+^ T cells by catalyzing K63‐linked ubiquitination at K233, thereby antagonizing K48‐linked ubiquitination and degradation. This modification maintains high PD‐1 abundance, dampens cytotoxic T cell activation, and promotes tumor immune evasion. Genetic ablation of Trim21 reduces PD‐1 levels, restores effector function, and enhances responsiveness to anti‐CTLA‐4 therapy, while also augmenting CAR‐T cell antitumor efficacy [51]. On the contrary, kelch like family member 22 (KLHL22), an adaptor of the cullin 3 (CUL3)–ring‐box 1 (RBX1) E3 ligase complex, preserves T cell function by mediating K48‐linked ubiquitination and proteasomal degradation of incompletely glycosylated PD‐1 before its surface transport. Loss of KLHL22 leads to PD‐1 overaccumulation, excessive T cell suppression, and accelerated tumor progression, while colorectal cancer–infiltrating T cells exhibit markedly reduced KLHL22 expression. Mechanistically, PD‐1 ubiquitination occurs predominantly at K210 and K233, with immature PD‐1 showing higher ubiquitination and faster turnover than mature PD‐1, highlighting a proteostatic checkpoint that safeguards PD‐1 homeostasis in antitumor immunity (Figure 3B) [52]. Moreover, polypyrimidine region‐ binding protein 3 (PTBP3) drives exon skipping of IL‐18 to generate the tumor‐specific ΔIL‐18 isoform, which orchestrates immune evasion in gallbladder cancer. ΔIL‐18 suppresses f‐box protein 38 (FBXO38) transcription in CD8^+^ T cells, reducing PD‐1 ubiquitination and degradation to sustain inhibitory signaling and weaken cytotoxic activity. The histone mark H3K36me3 recruits PTBP3 through MRG15 to promote IL‐18 exon skipping, while SET domain containing 2 (SETD2)‐mediated interaction with hnRNPL disrupts this process, revealing an integrated chromatin–splicing mechanism that links epigenetic modification to impaired T cell–mediated immunity [53]. Interestingly, PD‐1 not only undergoes multilayered post‐translational regulation but also reciprocally reshapes the PTM landscape that governs T cell signaling. PD‐1 engagement induces BATF‐dependent expression of the E3 ligase membrane associated ring‐CH‐type finger 5 (MARCH5), which catalyzes K27‐linked ubiquitination and lysosomal degradation of the common γ chain (γ_c_) receptor, thereby dampening cytokine signaling. Concurrently, src‐homology 2 domain‐containing protein tyrosine phosphatase 2 (SHP‐2) activation dephosphorylates γ_c_^Y357^, further suppressing IL‐2 responsiveness. PD‐1 blockade restores γ_c_ stability and cytokine sensitivity, while pharmacologic inhibition of MARCH5 amplifies IL‐2–driven CD8^+^ T cell activation, unveiling a regulatory axis that fine‐tunes effector function and therapeutic responsiveness [54].

Beyond checkpoint molecules, additional E3 ligases modulate co‐stimulatory and metabolic pathways critical for CD8^+^ T cell performance. The CD134 (OX40)–TNF receptor associated factor 6 (TRAF6) signaling axis enhances CD8^+^ T cell immunity by inducing K63‐linked ubiquitination and lysosomal degradation of CTLA‐4. TRAF6, through its RING domain, directly promotes CTLA‐4 turnover in a T cell–intrinsic manner, relieving inhibitory signaling and amplifying effector activity. Activation of OX40 further potentiates this pathway, establishing OX40–TRAF6–mediated CTLA‐4 degradation as a key mechanism that strengthens antitumor responses and improves the efficacy of checkpoint‐based immunotherapy [55]. Similarly, Pellino1 (Peli1) functions as an E3 ligase that suppresses CD8^+^ T cell effector activity by promoting K48‐linked ubiquitination and degradation of protein kinase C‐theta (PKCθ), a pivotal kinase in TCR signaling. Loss of Peli1 stabilizes PKCθ, sustaining downstream activation cascades that enhance cytokine production, prevent dysfunction, and maintain hyperactivated antitumor states in tumor‐infiltrating CD8^+^ T cells [56]. Moreover, tumor‐derived exosomal β‐TrCP drives CD8^+^ T cell dysfunction by catalyzing ubiquitination and degradation of yes1 associated transcriptional regulator (YAP1), thereby repressing transcription of amino acid transporters solute carrier family 7 member 5 (SLC7A5) and solute carrier family 38 member 1 (SLC38A1). This disruption of amino acid uptake leads to mTOR inactivation, metabolic insufficiency, and loss of effector function. Blocking β‐TrCP restores transporter expression, reinvigorates CD8^+^ T cell metabolism, and enhances antitumor immunity [57]. E3 ligases also intersect with metabolic checkpoints that fine‐tune mTORC1 activity. Peli1 restrains T cell metabolic reprogramming by catalyzing non‐degradative ubiquitination of TSC complex subunit 1 (TSC1), which stabilizes the TSC1–TSC complex subunit 2 (TSC2) complex and enforces suppression of mTORC1. This post‐translational control dampens glycolysis and limits the metabolic fitness of both CD4^+^ and CD8^+^ T cells, thereby curbing their infiltration and antitumor activity. Ablation of Peli1 disrupts this brake, unleashing mTORC1‐driven metabolic activity and markedly enhancing T cell–mediated tumor rejection [58].

#### 3.1.2 DUB‐Mediated Regulation of CD8^+^ T Cell Function in Cancer

Deubiquitinases (DUBs), traditionally linked to protein homeostasis and tumorigenesis, also govern CD8^+^ T cell stability and functional adaptability in the TME. By reversing E3 ligase–mediated ubiquitination, these enzymes fine‐tune receptor turnover, metabolic fitness, and chromatin accessibility, thereby shaping the balance between activation and dysfunction [59, 60]. Beyond receptor‐ and metabolic‐specific DUBs, additional enzymes regulate protein turnover and mitochondrial integrity to sustain CD8^+^ T cell lineage stability and prevent immune escape. Notably, the DUB ubiquitin specific peptidase 1 (USP1) interacts with and stabilizes Id2 and Id3 during T cell activation, linking protein turnover to lineage maintenance. While dispensable for primary effector differentiation, USP1 is essential for sustaining memory CD8^+^ T cell persistence and robust recall expansion by preserving Id2 abundance. Its loss compromises long‐term cytotoxic capacity, revealing a protein stability checkpoint that safeguards memory T cell renewal and immune durability [61]. By contrast, USP30 inhibits parkin‐mediated mitophagy and promotes mitochondrial transfer. It is highly expressed in cancer cells and transferred to tumor‐infiltrating lymphocytes (TILs) along with mitochondria. In TILs, USP30 prevents mitophagy, allowing mutated mitochondrial DNA to persist, which contributes to immune evasion [62]. Ubiquitin specific peptidase 24 (USP24), induced by IL‐6–activated STAT3 signaling, removes K48‐linked ubiquitin chains from PD‐1, preventing its degradation and sustaining inhibitory signaling. Stabilized PD‐1 enforces exhaustion and weakens cytotoxicity, whereas USP24 inhibition reduces PD‐1 abundance, restores effector function, and synergizes with CTLA‐4 blockade to suppress tumor growth. Elevated USP24 in tumor‐infiltrating CD8^+^ T cells correlates with immune dysfunction and poor immunotherapy response, defining it as a key regulator of PD‐1 persistence [63]. Similarly, ubiquitin specific peptidase 7 (USP7) maintains mitochondrial stability by deubiquitinating C‐X9‐C motif containing 1 (CMC1), a complex IV chaperone whose stabilization in lactate‐rich tumor environments promotes terminal differentiation and dysfunction. In contrast, Cmc1 deficiency preserves memory‐like, metabolically quiescent CD8^+^ T cells resistant to dysfunction, positioning the USP7–CMC1 axis as an immunometabolic checkpoint linking ubiquitin signaling to T cell fate (Figure 3C) [64]. At the epigenetic level, ASXL transcriptional regulator 1 (ASXL1), a cofactor of the polycomb repressive deubiquitinase (PR‐DUB) complex, modulates H2AK119 ubiquitination to preserve chromatin accessibility and sustain a progenitor‐exhausted population with self‐renewal and antitumor potential. Loss of Asxl1 enhances CD8^+^ T cell persistence and synergizes with PD‐L1 blockade to achieve durable tumor control [65]. Together, these findings highlight that distinct DUBs—from canonical USP family enzymes to chromatin‐associated PR‐DUB complexes—converge to stabilize key signaling and metabolic nodes that determine CD8^+^ T cell longevity and functional equilibrium in cancer.

The dynamic interplay between ubiquitination and deubiquitination acts as a finely tuned regulatory circuit rather than a simple on–off switch, enabling CD8^+^ T cells to adjust receptor signaling, metabolic flux, and differentiation trajectories in response to the evolving tumor milieu. Disruption of this balance—whether through excessive ubiquitin conjugation or insufficient deubiquitination—distorts signaling thresholds, destabilizes metabolic homeostasis, and accelerates the progression toward dysfunction or exhaustion. Notably, inhibitory receptors such as PD‐1 not only undergo multilayered post‐translational regulation but also reciprocally reshape the modification landscape of key signaling molecules, establishing feedback loops that reinforce inhibitory signaling and functional decline. From a translational perspective, the ubiquitin–deubiquitinase axis constitutes a bidirectional control system that maintains CD8^+^ T cell plasticity and functional equilibrium under chronic antigenic stress. Therapeutically tuning this axis may offer a strategy to reprogram exhausted states, restore effector competence, and prolong the efficacy of immune checkpoint–based cancer immunotherapy.

### 3.2 NEDDylation

**FIGURE 4:**
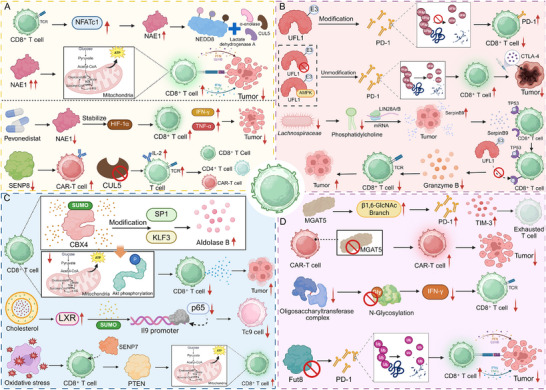
Core regulatory mechanisms of PTMs in CD8^+^ T cell–mediated antitumor immunity. (A) NEDDylation. T cell receptor signaling activates NFATc1, inducing expression of the NEDD8‐activating enzyme NAE1. NEDDylation, mainly through Cullin‐RING ligase complexes, modulates metabolic regulators to sustain glycolysis and oxidative phosphorylation, thereby enhancing CD8^+^ T cell metabolic fitness and cytotoxicity. Increased NAE1 activity strengthens antitumor immunity, whereas inhibition by pevonedistat stabilizes HIF‐1α, promotes cytokine production, and facilitates tumor infiltration. Inhibition of SENP8 enhances CAR‐T cell cytotoxicity, and CUL5 deletion favors effector differentiation. (B) UFMylation. UFL1‐mediated UFMylation contributes to PD‐1 stabilization and restrains CD8^+^ T cell activity. Loss of UFL1 or AMPK‐dependent phosphorylation of UFL1 promotes PD‐1 degradation, enhancing T cell activation and sensitivity to CTLA‐4 blockade. Reduced Lachnospiraceae abundance decreases phosphatidylcholine production, promoting LIN28A/B‐dependent maturation and tumor‐derived exosomal secretion of SerpinB9, which interferes with TP53‐dependent UFMylation, reduces granzyme B expression, and impairs cytotoxicity. (C) SUMOylation. Nuclear CBX4 enhances aldolase B expression via SUMOylation‐dependent regulation of SP1 and KLF3, suppressing Akt phosphorylation, glycolysis, and ATP production. Cholesterol‐induced SUMOylation of liver X receptor inhibits p65 binding to the Il9 promoter, limiting Tc9 cell persistence. Oxidative stress–induced cytoplasmic translocation of SENP7 destabilizes PTEN, relieves metabolic constraints, and enhances CD8^+^ T cell effector function. (D) Glycosylation. MGAT5‐mediated β1,6‐GlcNAc branching upregulates PD‐1 and TIM‐3 expression, promoting T cell exhaustion, whereas MGAT5 deletion restores effector function. Impaired N‐glycosylation caused by oligosaccharyltransferase downregulation disrupts cytokine secretion and receptor stability. Deletion of Fut8 promotes PD‐1 ubiquitination and degradation, enhancing CD8^+^ T cell activation and tumor clearance. Created in https://BioRender.com.

NEDDylation, a ubiquitin‐like PTM, has emerged as a critical regulator of T cell activation, proteostasis, and metabolic adaptation. By conjugating neural precursorcell‐expressed developmentally downregulated 8 (NEDD8) to specific substrates, this pathway coordinates signaling and energy metabolism to determine the activation state and longevity of CD8^+^ T cells in tumors [66, 67]. For instance, NEDDylation governs CD8^+^ T cell activation and metabolic fitness by modifying key glycolytic enzymes, including lactate dehydrogenase A, α‐enolase, and hexokinase 1, thereby sustaining glycolysis and oxidative phosphorylation. Loss of the NEDD8 activating enzyme E1 subunit 1 (NAE1) impairs effector differentiation and antitumor function, whereas inhibition of the deNEDDylase SENP8 enhances CAR‐T cytotoxicity. Through metabolic reprogramming, NEDDylation establishes the bioenergetic foundation necessary for CD8^+^ T cell effector function and durable antitumor immunity (Figure 4A) [68]. Likewise, TCR engagement activates NFATc1‐dependent induction of NAE1, driving neddylation that sustains the proteomic and metabolic programs essential for CD8^+^ T cell activation. Loss of NAE1 disrupts mitochondrial function, protein synthesis, and proliferation, leading to defective effector differentiation and diminished antitumor activity. Conversely, enforced NAE1 expression enhances the metabolic fitness and cytotoxic potential of tumor‐infiltrating CD8^+^ T cells [69].

On the contrary, inhibition of the NEDD8‐activating enzyme by pevonedistat reprograms CD8^+^ T cells toward a proinflammatory and cytotoxic state characterized by elevated TNFα and IFNγ production. By suppressing Cullin‐RING E3 ligase (CRL) activity, pevonedistat stabilizes HIF‐1α, amplifying interferon and chemokine signaling to enhance tumor infiltration and immune activation. In lymphoid malignancies, this restoration of T cell function delays tumor progression, while combination with PD‐1 blockade synergistically augments antitumor efficacy [70]. Similarly, cullin 5 (CUL5), activated through neddylation, assembles an E3 ligase complex with PCMTD2 to restrain TCR and IL‐2 signaling in CD8^+^ T cells. Deletion of CUL5 disrupts this inhibitory axis, leading to broad proteomic reprogramming that enhances cytokine responsiveness, effector differentiation, and tumor control. Pharmacologic blockade of neddylation mimics these effects, while CTLA‐4 inactivation further amplifies antitumor potency [71]. These findings highlight the context‐dependent duality of NEDDylation in CD8^+^ T cells. While transient NEDDylation supports metabolic and effector programming, sustained or dysregulated activity can reinforce inhibitory signaling and dysfunction. Fine‐tuning NEDDylation dynamics may thus balance metabolic fitness with controlled activation to improve antitumor immunity.

### 3.3 UFMylation

UFMylation, a newly recognized ubiquitin‐like modification, has emerged as a pivotal regulator of protein quality control and immune signaling. The UFMylation E3 ligase UFM1‐specific ligase 1 (UFL1) sustains PD‐1 stability in CD8^+^ T cells by modifying PD‐1 and preventing its ubiquitin‐mediated degradation. Loss of UFL1 diminishes PD‐1 UFMylation, leading to receptor destabilization and enhanced T cell activation. AMPK‐dependent phosphorylation of UFL1 at Thr536 disrupts its interaction with PD‐1, further promoting PD‐1 turnover. UFL1 deficiency thereby amplifies cytotoxic T cell infiltration and strengthens antitumor immunity, sensitizing tumors to CTLA‐4 blockade (Figure 4B) [72]. Interestingly, microbiota‐derived phosphatidylcholine orchestrates CD8^+^ T cell suppression through a lipid–protein regulatory circuit. Reduced *Lachnospiraceae* abundance diminishes phosphatidylcholine, which in turn promotes exosomal serpin family B member 9 (SerpinB9) production from tumor cells via LIN28A/B‐dependent mRNA maturation. Exosomal SerpinB9 binds Tumor protein 53 (TP53) in CD8^+^ T cells, preventing its interaction with the ubiquitin fold modifier 1 (UFM1) conjugation enzyme and thereby reducing TP53 UFMylation and granzyme B expression. This cascade inactivates cytotoxic T cells, revealing a microbiota‐lipid‐UFMylation axis that remodels immune surveillance in multiple myeloma [73].

### 3.4 SUMOylation

SUMOylation, a reversible PTM analogous to ubiquitination, fine‐tunes protein localization, stability, and transcriptional control through the covalent conjugation of SUMO to lysine residues. In CD8^+^ T cells, SUMOylation and deSUMOylation collectively shape activation thresholds, metabolic fitness, and effector differentiation in response to the TME [74, 75, 76]. Notably, ubiquitin conjugating enzyme 9 (UBC9)‐dependent SUMO conjugation is indispensable for thymocyte maturation and CD8^+^ T cell survival. Loss of *Ubc9* impairs late‐stage development following positive selection, leading to reduced CD4^+^ and CD8^+^ single‐positive populations due to defective proliferation and enhanced apoptosis. Mechanistically, SUMO signaling maintains IL‐7–driven survival pathways and regulates nuclear factor of activated T cells (NFAT) nuclear retention upon TCR activation. Disruption of this modification compromises IL‐7 responsiveness and transcriptional stability, underscoring UBC9 as a central regulator linking nuclear signaling dynamics to CD8^+^ T cell maturation and homeostasis [77]. Additionally, modification of RORγt at lysine 31 by SUMO3 governs T cell lineage programming through recruitment of the coactivator complex lysine acetyltransferase 2A (KAT2A)–steroid receptor coactivator 1 (SRC1), enhancing its transcriptional activity. Disruption of this modification impairs Th17 differentiation, delays maturation of thymic CD8^+^ immature single‐positive cells, and alters lymphoid organogenesis [78]. Chromobox 4 (CBX4) restrains CD8^+^ T cell antitumor activity by enforcing a glycolytic blockade through SUMO‐dependent transcriptional control. Within tumor‐infiltrating T cells, CBX4 modifies specificity protein 1 (SP1) and kruppel‐like factor 3 (KLF3) to elevate Aldolase B expression, which in turn suppresses AKT phosphorylation, glycolysis, and ATP generation. This metabolic repression diminishes CD8^+^ T cell effector capacity, while CBX4 deficiency restores metabolic vigor and enhances responsiveness to PD‐1 blockade. By coupling SUMO‐driven transcriptional regulation to metabolic signaling, CBX4 emerges as a key brake on CD8^+^ T cell function within the TME (Figure 4C) [79]. In addition, cholesterol constrains IL‐9–producing CD8^+^ T cell differentiation and antitumor function through activating liver X receptor (LXR)‐dependent signaling. Elevated cholesterol or its metabolites activate LXRs, inducing their SUMO modification and diminishing p65 recruitment to the Il9 promoter, thereby suppressing IL‐9 transcription. This repression limits Tc9 cell persistence and effector activity, while cholesterol depletion restores IL‐9 expression and enhances tumor control [80]. Notably, TAK‐981 inhibits the SUMO‐activating enzyme, blocking the conjugation of SUMO proteins to target substrates that regulate transcriptional control, DNA repair, and signaling in T cells. This disruption triggers type I IFN responses while preserving TCR‐driven activation, marked by increased CD69 and CD38 expression. In chronic lymphocytic leukemia‐derived T cells, impaired SUMOylation reduces Treg differentiation and enhances IFN‐γ secretion by both CD4^+^ and CD8^+^ subsets. Mouse models further confirm that inhibition of SUMO modification reprograms T cell responses in an evolutionarily conserved manner [81]. Complementing this, deSUMOylation pathways safeguard T cell metabolic adaptability under oxidative stress. SUMO specific peptidase 7 (SENP7) maintains CD8^+^ T cell metabolic fitness and effector function by sensing oxidative stress within the TME. Upon reactive oxygen species accumulation, SENP7 translocates to the cytosol and mediates PTEN deSUMOylation, promoting its degradation and preventing PTEN‐driven metabolic suppression. SENP7 deficiency impairs both glycolysis and oxidative phosphorylation, leading to reduced proliferation and diminished antitumor capacity [82]. Furthermore, glucose restriction enhances CD8^+^ T cell memory formation by engaging AMPK–sentrin‐specific protease 1 (SENP1)–sirtuin 3 (Sirt3) signaling within mitochondria. AMPK activation triggers SENP1‐dependent deSUMOylation of Sirt3, augmenting its deacetylase activity and promoting oxidative phosphorylation and mitochondrial fusion. Activated Sirt3 reduces acetylation of the metalloprotease YME1 like 1 ATPase (YME1L1), limiting OPA1 mitochondrial dynamin like GTPase (OPA1) cleavage and sustaining mitochondrial integrity to support T cell survival and memory development. Conversely, the glycolytic metabolite fructose‐1,6‐bisphosphate suppresses this AMPK–SENP1–Sirt3 axis, linking metabolic limitation to mitochondrial remodeling and long‐lived CD8^+^ T cell functionality [83].

### 3.5 Glycosylation

Glycosylation modulates CD8^+^ T cell phenotype by organizing receptor architecture and regulating signaling, trafficking, and stability, thereby balancing activation, dysfunction, and persistence within the TME. Among glycosylation pathways, O‐glycan modifications critically influence CD8^+^ T cell effector potential and cytokine production. Notch signaling modulates CD8^+^ T cell activation through GCNT1‐driven O‐glycosylation of the sialomucin CD43. Induction of GCNT1 enhances core‐2 O‐glycan formation on CD43, marking T cells with increased cytokine production and effector potential [84]. Specifically, in the context of N‐glycan remodeling, branched N‐glycan remodeling orchestrated by alpha‐1,6‐mannosylglycoprotein 6‐beta‐N‐acetylglucosaminyltransferase (MGAT5) shapes CD8^+^ T cell fate within tumors. Elevated β1,6‐GlcNAc branching promotes an exhausted phenotype characterized by heightened PD‐1 and TIM‐3 expression, restraining cytotoxicity. Disruption of MGAT5 reverses this program, restoring effector activity and enhancing tumor cell killing. In engineered CAR‐T cells, MGAT5 deletion similarly boosts antitumor efficacy in solid tumors (Figure 4D) [85]. Similarly, progressive branching of N‐glycans in colonic T cells drives immune suppression and tumor progression during colitis‐associated colorectal cancer. Enhanced MGAT5‐mediated glycan branching imposes inhibitory signaling that limits CD8^+^ and γδ T cell infiltration, weakening antitumor immunity from inflammation to malignancy. Genetic ablation of this glycan pathway restores effector T cell activity and suppresses tumor formation. Clinically, elevated branched N‐glycosylation in inflamed inflammatory bowel disease (IBD) lesions predicts cancer risk, establishing T cell glycan remodeling as both a mechanistic driver and a potential biomarker of colitis‐associated tumorigenesis [86]. On the contrary, loss of N‐glycan transfer in tumor‐infiltrating CD8^+^ T cells disrupts IFN‐γ–driven effector programs and accelerates dysfunction. Downregulation of the oligosaccharyltransferase complex within the TME compromises N‐glycosylation, leading to impaired cytokine production and weakened antitumor immunity. Restoration of this complex reinstates IFN‐γ secretion and revives cytotoxic function, curbing tumor progression. These apparently contradictory observations highlight the biphasic and context‐dependent nature of N‐glycosylation in regulating CD8^+^ T cell function [87]. Excessive MGAT5‐mediated β1,6‐GlcNAc branching reinforces inhibitory receptor clustering and galectin lattice formation, strengthening exhaustion‐associated signaling. Conversely, insufficient N‐glycan transfer impairs receptor folding, trafficking, and cytokine responsiveness, thereby weakening effector differentiation. Both extremes ultimately converge on diminished T cell competence—one through signaling suppression, the other via structural instability. Collectively, these findings reveal that precise glycosylation homeostasis is essential to maintain receptor integrity, signaling plasticity, and sustained antitumor immunity within the metabolically restrictive TME.

Importantly, glycosylation serves as a critical post‐translational determinant of PD‐1 stability, trafficking, and surface abundance on CD8^+^ T cells. For instance, Rab37 orchestrates PD‐1 trafficking and membrane localization in CD8^+^ T cells, sustaining dysfunction within the TME. In its GTP‐bound form, Rab37 directs PD‐1‐containing vesicles to the plasma membrane, whereas disruption of PD‐1 glycosylation impairs vesicular recruitment and surface presentation. Loss of Rab37 restores T cell proliferation and cytotoxicity, while elevated Rab37–PD‐1–TIM3 expression in tumor‐infiltrating T cells correlates with advanced disease and poor survival [88]. Additionally, loss of core fucosylation destabilizes PD‐1 on CD8^+^ cytotoxic T lymphocytes by enhancing its ubiquitination and proteasomal degradation. Fut8 deficiency reduces PD‐1 surface expression, thereby augmenting T cell activation, cytotoxicity, and tumor clearance. These findings reveal glycosylation‐dependent control of PD‐1 protein stability as a post‐translational mechanism that can be exploited to potentiate antitumor immunity [89]. In parallel, the immune‐suppressive activity of PD‐L1 is tightly regulated by glycosylation. GSK3β phosphorylates PD‐L1, promoting its degradation via β‐TrCP, but glycosylation at N192, N200, and N219 prevents GSK3β binding, thereby stabilizing PD‐L1. In basal‐like breast cancer, EGF inactivates GSK3β, further enhancing PD‐L1 stability. Inhibition of EGF signaling with gefitinib destabilizes PD‐L1, enhancing T cell‐mediated antitumor immunity and improving the efficacy of PD‐1 blockade in mouse models [90].

### 3.6 Phosphorylation

**FIGURE 5:**
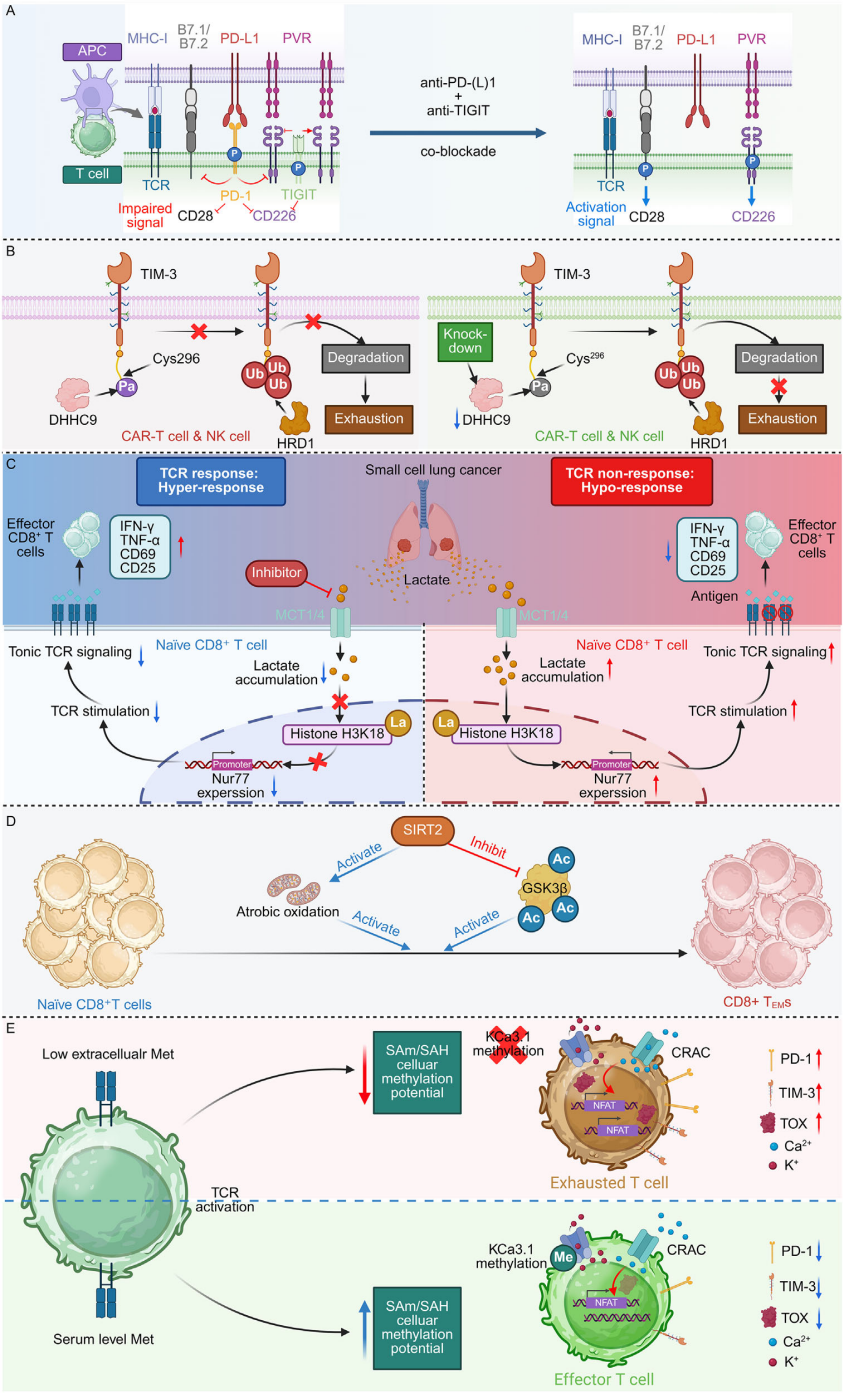
PTM–dependent control of inhibitory signaling, metabolism, and fate decisions in CD8^+^ T cells. (A) Phosphorylation‐dependent regulation of costimulatory signaling by immune checkpoints. PD‐1 suppresses CD28 and CD226 signaling through its intracellular ITIM‐mediated inhibition of phosphorylation, whereas TIGIT limits CD226 activation by preventing its engagement with CD155. In tumor‐infiltrating CD8^+^ T cells, co‐expression of CD28 and CD226 defines a population with high proliferative and effector potential that is functionally restrained by PD‐1 and TIGIT. Dual blockade is required to fully restore CD226 signaling and optimal effector function. (B) Palmitoylation‐dependent stabilization of TIM‐3 in exhausted lymphocytes. DHHC9‐mediated palmitoylation of TIM‐3 at Cys296 prevents HRD1‐mediated ubiquitination and proteasomal degradation, sustaining surface TIM‐3 expression and promoting T cell exhaustion. Genetic or pharmacological disruption of this palmitoylation accelerates TIM‐3 turnover and restores CAR‐T cell and NK cell cytotoxicity, highlighting lipid modification–driven receptor stabilization as a mechanism of immune suppression. (C) Lactate‐driven histone lactylation programs TCR hyporesponsiveness. In small‐cell lung cancer, lactate accumulation induces histone H3K18 lactylation in naïve CD8^+^ T cells, promoting Nur77 expression and persistent tonic TCR signaling, which impairs antigen responsiveness and effector function. Inhibition of lactate production reverses this epigenetic program and enhances sensitivity to PD‐1 blockade. (D) Acetylation‐dependent metabolic control of effector memory differentiation. SIRT2 promotes oxidative metabolism and effector memory CD8^+^ T cell formation by restraining acetylation of GSK3β and coordinating metabolic signaling. Loss of SIRT2 disrupts this balance, favoring naïve phenotypes and weakening antitumor immunity. (E) Methionine‐dependent methylation as an early fate‐determining checkpoint. During the initial minutes of TCR activation, extracellular methionine availability controls cellular methylation potential and arginine methylation of the potassium channel KCa3.1, modulating calcium signaling, NFAT activity, and downstream transcriptional programs. This temporally restricted methylation circuit imprints long‐term effector vs. exhausted CD8^+^ T cell fates. Created in https://BioRender.com.

Phosphorylation serves as a central post‐translational mechanism that rapidly translates extracellular cues into functional outputs in CD8^+^ T cells. Elevated expression of inhibitory receptors and tumor‐associated regulators imposes phosphorylation‐dependent suppression on T cell signaling [91, 92]. For instance, PD‐1 directly inhibits the phosphorylation of CD226 and CD28 through its intracellular immunoreceptor tyrosine‐based inhibitory motif (ITIM) domain, while T cell immune receptor with Ig and ITIM domains (TIGIT) blocks CD226 engagement with its ligand CD155. In tumor‐infiltrating CD8^+^ T cells, co‐expression of CD226 and CD28 marks a population poised for expansion, but their signaling is restrained by PD‐1 and TIGIT. Full restoration of CD226 activity—and thus optimal CD8^+^ T cell effector function—requires dual blockade, highlighting the phosphorylation‐dependent control of costimulator (Figure 5A) [93]. Similarly, elevated B7‐H4 expression correlates with reduced CD8^+^ T cell density and decreased production of IFN‐γ and TNF‐α, alongside diminished CD137 and CD103 expression. Mechanistically, B7‐H4 inhibits AKT and eNOS phosphorylation, inducing early T cell dysfunction and limiting antitumor immunity [94]. Moreover, BCL9 transcription coactivator (BCL9) suppression enhances CD8^+^ T cell infiltration and response to anti‐PD‐1 therapy by reshaping tumor–immune interactions. Mechanistically, BCL9 inhibition increases vav guanine nucleotide exchange factor 1 (VAV1) phosphorylation in CD8^+^ T cells and induces GLI family zinc finger 1 (GLI1) and PATCH expression to upregulate CD155 on tumor cells, altering CD155–CD226/CD96 signaling. High BCL9 expression correlates with reduced CD226 and poor clinical outcomes, particularly in patients with adenomatous polyposis coli mutations [95]. Valosin‐containing protein (VCP) impairs CD8^+^ T cell function in hepatocellular carcinoma by promoting glycerol‐3‐phosphate dehydrogenase 1 like (GPD1L)‐mediated accumulation of glycerol‐3‐phosphate, which interacts with LCK and increases phosphorylation at Tyr505, an inhibitory site. This disrupts TCR signaling and suppresses T cell activation and cytotoxicity. Targeting VCP restores T cell function and enhances anti‐PD1 efficacy, offering a strategy to overcome immune suppression in the TME [96]. Notably, sialic acid‐binding immunoglobulin‐like lectin 9 (Siglec‐9) marks a tumor‐resident effector memory CD8^+^ T cell subset in melanoma and functions as a glycan‐dependent inhibitory checkpoint. Engagement of Siglec‐9 by tumor‐expressed ligands triggers src‐homology 2 domain‐containing protein tyrosine phosphatase 1 (SHP‐1) phosphorylation, suppressing TCR signaling, cytokine release, and cytotoxic activity. This interaction confines T cell activation within the TME, dampening antitumor immunity [97]. By contrast, cytokine‐mediated phosphorylation reinforces T cell persistence and effector function. IL‐21 enhances the antitumor efficacy of AFP‐specific TCR‐T cells in hepatocellular carcinoma by promoting STAT3 phosphorylation, which supports proliferation, memory differentiation, and reduced PD‐1 expression and apoptosis. Engineering TCR‐T cells with a synthetic IL‐21 receptor enables ligand‐independent STAT3 activation, further improving persistence and cytotoxicity upon repetitive antigen exposure [98].

Importantly, metabolic and microbial inputs provide additional means of modulating phosphorylation‐driven activation. The microbial metabolite butyrate reprograms CD8^+^ T cells toward a dual effector–memory phenotype by enhancing mTOR phosphorylation, TCR expression, and IFN‐γ production while limiting excessive proliferation. Upon transfer, butyrate‐conditioned CD8^+^ T cells accumulate within tumors, sustain cytotoxic cytokine secretion, and enrich for IL‐15Rβ^+^ T‐bet^+^ effector‐memory clusters, leading to superior tumor control [99]. L‐selenomethionine enhances CD8^+^ T cell effector function by promoting LCK phosphorylation, leading to strengthened TCR signaling. Oral administration suppresses colorectal tumor growth and increases antitumor immunity, highlighting a microbiota‐derived metabolite that potentiates T cell activity through modulation of intracellular signaling dynamics [100]. Asparagine enhances CD8^+^ T cell activation and antitumor function by directly modulating LCK activity. Rather than altering metabolic flux, asparagine binds to LCK and promotes its phosphorylation at Tyr394 and Tyr505, leading to amplified TCR signaling. Increased asparagine availability boosts effector cytokine production and cytotoxicity, while depletion or blockade of asparagine uptake impairs CD8^+^ T cell responses [101].

Pharmacologic agents can similarly rewire phosphorylation networks to restore antitumor immunity. Astragalus polysaccharide (APS) enhances the persistence and antitumor activity of glypican 3 (GPC3)‐targeted CAR‐T cells in hepatocellular carcinoma by promoting the expansion of CD122^+^/CXCR3^+^/PD‐1^−^ memory T cells. APS augments CAR‐T cell proliferation, migration, and effector function, while upregulating CXCL9/10 in tumors. Mechanistically, APS activates STAT5 phosphorylation, and blockade of this pathway abrogates its effects, highlighting a key signaling axis in modulating CAR‐T cell efficacy [102]. Tetracyclines enhance antitumor T cell immunity by activating early TCR signaling cascades. Minocycline treatment increases Zap70 phosphorylation and upregulates CD69 and nuclear receptor subfamily 4, group A, member 1 (Nur77) expression in CD8^+^ T cells, thereby augmenting granzyme B production and interferon‐γ secretion. These effects strengthen cytotoxic activity against tumor cells in both human and murine models, independent of PD‐1 blockade [103].

### 3.7 Palmitoylation

Palmitoylation, a reversible lipid modification involving the covalent attachment of palmitic acid to cysteine residues, critically regulates protein stability, membrane localization, and signaling in CD8^+^ T cells within tumors. Dysregulation of palmitoylation in the TME contributes to immune suppression and therapeutic resistance through coordinated effects on both tumor metabolism and immune checkpoint control [104, 105]. Loss of the E3 ubiquitin ligase Riplet in hepatocellular carcinoma enhances fatty acid synthase (FAS) stability, driving excess fatty acid production that induces CD8^+^ T cell dysfunction. Tumor‐derived palmitic acid activates STAT3 through enhanced palmitoylation, reinforcing terminal T cell dysfunction and resistance to anti‐PD‐1 therapy. Inhibition of fatty acid synthesis restores immune responsiveness, linking metabolic dysregulation to impaired T cell function in Riplet‐deficient tumors [106]. Concurrently, TIM‐3 stability and immune inhibitory function are governed by ZDHHC palmitoyltransferase 9 (DHHC9)‐mediated palmitoylation at Cys^296^. This lipid modification prevents HRD1 binding, suppressing ubiquitination and degradation, thereby sustaining surface TIM‐3 expression and promoting T cell dysfunction. Disruption of DHHC9 or blockade of TIM‐3 palmitoylation accelerates its turnover, restoring CAR‐T and NK cell activity. Elevated DHHC9 correlates with TIM‐3 accumulation and poor prognosis in hepatocellular carcinoma, highlighting palmitoylation‐driven stabilization as a key mechanism restraining antitumor immunity (Figure 5B) [107].

### 3.8 Lactylation

Lactate‐derived histone lactylation integrates cellular metabolism with transcriptional regulation in CD8^+^ T cells. Enrichment of H3K18la and H3K9la activates gene networks governing effector differentiation and metabolic reprogramming, linking glycolytic flux to functional adaptation. Distinct lactylation patterns define T cell subsets according to their energetic states, while modulation of these marks through metabolic or epigenetic interventions reshapes cytokine production and antitumor activity. However, excessive or context‐specific accumulation of lactate can reprogram histone lactylation toward immunosuppressive outcomes [108, 109]. Lactate accumulation in small cell lung cancer drives immune evasion through histone H3K18 lactylation–dependent induction of the transcription factor Nur77 in naïve CD8^+^ T cells. This modification triggers persistent TCR signaling and transcriptional reprogramming that impair antigen recognition and cytotoxic function. Suppression of lactate production abrogates H3K18la‐mediated Nur77 activation, restoring CD8^+^ T cell responsiveness and potentiating PD‐1 blockade efficacy (Figure 5C) [110]. Similarly, enhanced glycolysis in obesity amplifies lactate flux and histone lysine lactylation in CD8^+^ T cells via monocarboxylate transporter 1 (MCT1)‐mediated transport, leading to elevated PD‐1 expression and altered activation dynamics. This metabolic‐epigenetic remodeling sensitizes T cells to checkpoint inhibition, improving anti–PD‐1 efficacy in obese hosts. In tumor co‐culture models, glycolytic or lactate enrichment recapitulates these effects, demonstrating that glycolytic lactylation serves as a metabolic rheostat linking systemic energy status to CD8^+^ T cell responsiveness during immunotherapy [111].

### 3.9 Acetylation

Acetylation is a reversible PTM in which an acetyl group is transferred to lysine residues on histone or non‐histone proteins, thereby modulating protein activity, stability, and chromatin accessibility. This process is dynamically controlled by lysine acetyltransferases (KATs) and deacetylases (KDACs/Sirtuins), enabling cells to rapidly adjust transcriptional programs and metabolic pathways in response to environmental cues [112]. For instance, SIRT2 governs CD8^+^ T cell differentiation and effector memory formation through acetylation‐dependent control of metabolic signaling. Reduced SIRT2 expression in breast cancer correlates with diminished effector memory and increased naïve T cell populations. Mechanistically, SIRT2 enhances oxidative metabolism while restraining GSK3β acetylation, thereby promoting the transition toward an effector memory phenotype. Loss of SIRT2 disrupts this balance, weakening CD8^+^ T cell function and antitumor immunity, highlighting its pivotal role in coupling metabolic fitness to differentiation within the tumor immune response (Figure 5D) [113]. Notably, the active metabolite 1α,25(OH)_2_D_3_ restores cytotoxic T cell function by epigenetically and transcriptionally reprogramming dysfunction‐associated signaling. Upon ligand binding, nuclear translocation of the vitamin D receptor represses programmed cell death 1 (PDCD1), TIGIT, and TIM‐3 transcription while promoting CpG methylation of the PDCD1 promoter and H3K27 acetylation at the CD28 locus. This coordinated chromatin remodeling downregulates PD‐1, TIM‐3, and TIGIT while enhancing CD28 expression and Ca^2^^+^‐dependent Th1 cytokine production [114].

### 3.10 Methylation

Methylation is a widespread PTM involving the transfer of methyl groups to lysine or arginine residues on histone and non‐histone proteins, thereby influencing chromatin organization, transcriptional activity, and signaling dynamics. This modification is catalyzed by lysine and arginine methyltransferases and reversed by demethylases, generating a flexible and multilayered regulatory system [115, 116, 117]. Methionine (Met) availability during the earliest phase of CD8^+^ T cell activation serves as a decisive metabolic cue linking nutrient sensing to effector fate. Within 30 min of TCR engagement, extracellular Met fuels arginine methylation of the calcium‐activated potassium channel KCa3.1, modulating calcium flux and downstream transcriptional programs that determine dysfunction and antitumor potential. This temporally restricted methylation circuit endows T cells with long‐lasting functional imprints, revealing a metabolic checkpoint that couples nutrient dynamics to immune persistence and suggesting opportunities for precision metabolic intervention to enhance T cell immunity (Figure 5E) [118].

Beyond these rapid, metabolically coupled protein methylation events, stable DNA methylation programs impose long‐term epigenetic constraints on T cell fate, particularly under conditions of chronic antigen exposure and therapeutic T cell engineering. CAR‐T cell therapy is limited by dysfunction, characterized by the loss of multipotent potential during prolonged antigen exposure. Deleting DNMT3A in CAR‐T cells prevents dysfunction by preserving their proliferation and antitumor response. This is associated with interleukin‐10 upregulation and epigenetic silencing of key “stemness” genes, such as CD28, CCR7, TCF7, and LEF1. DNA methylation profiling provides an atlas of genes involved in CAR‐T cell dysfunction, offering insights into epigenetic regulation of immune cell fate and strategies to enhance CAR‐T cell efficacy in clinical applications [119].

## 4 Challenges and Future Directions in Deciphering and Targeting PTMs in CD8^+^ T Cells

**FIGURE 6:**
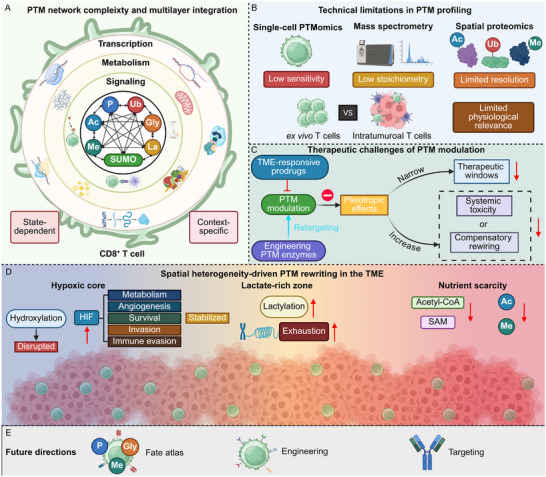
Challenges and future directions in deciphering and targeting post‐translational modification (PTM) networks in CD8^+^ T cells. (A) Network complexity and multilayer integration. PTMs form dense, state‐ and context‐dependent regulatory networks that integrate signaling, metabolism, and transcriptional control to govern CD8^+^ T cell fate decisions. Extensive crosstalk among phosphorylation, ubiquitination, acetylation, methylation, glycosylation, SUMOylation, and lactylation obscures causal relationships and complicates the identification of discrete therapeutic nodes. (B) Technical limitations in PTM profiling. Current approaches are constrained by low sensitivity and stoichiometric resolution, limited single‐cell compatibility, and insufficient spatial context, particularly when comparing ex vivo–expanded T cells with intratumoral populations. These limitations hinder accurate reconstruction of PTM dynamics across activation states and tumor niches. (C) Therapeutic challenges of PTM modulation. Because PTMs exert pleiotropic effects across interconnected pathways, global perturbation often narrows therapeutic windows and increases the risk of systemic toxicity or compensatory rewiring. Strategies such as TME–responsive prodrugs, retargeting or engineering of PTM enzymes, and cell‐specific delivery aim to enhance precision and context selectivity. (D) Spatial heterogeneity–driven PTM rewiring in the TME. Hypoxia, lactate accumulation, and nutrient scarcity impose region‐specific remodeling of PTM circuits, including disruption of hydroxylation–HIF axes, induction of lactylation‐associated exhaustion programs, and depletion of acetylation and methylation capacity, resulting in microregional divergence in CD8^+^ T cell functionality. (E) Future directions. Integrative PTM fate atlases, single‐cell and spatial PTMomics, genome and protein engineering, and targeted immunotherapeutic strategies are expected to enable rational, PTM‐informed modulation of CD8^+^ T cell responses and support the development of next‐generation precision immunotherapies. Created in https://BioRender.com.

As PTMs emerge as central regulators of CD8^+^ T cell activation, differentiation, and dysfunction, a new conceptual framework is taking shape: PTMs function as the key molecular interface connecting environmental cues, metabolic flux, and transcriptional circuitry to T cell fate determination. Yet this integrative capacity also underlies a distinctive set of scientific and translational challenges. PTMs operate within dense, multilayered regulatory networks, are highly dynamic and context‐dependent, and vary across spatially heterogeneous tumor niches. Moreover, PTM‐targeted interventions exert pleiotropic effects that complicate therapeutic precision. Addressing these challenges will require integrated technological, computational, and translational strategies capable of resolving PTM dynamics at high temporal, spatial, and functional resolution. Below, we outline the major obstacles and propose concrete solutions that could accelerate the development of PTM‐informed immunotherapies (Figure 6).

The complexity of PTM networks presents a major barrier to mechanistic resolution in CD8^+^ T cells. PTMs are embedded within tightly interconnected signaling, metabolic, and epigenetic layers, and their patterns vary substantially across differentiation states, tissue niches, and activation histories [120]. These signatures become even more heterogeneous under persistent TME pressures—such as hypoxia, chronic antigen stimulation, and elevated lactate—where PTM changes often reflect system‐wide circuit rewiring rather than isolated enzymatic events. This multidimensional integration obscures causal relationships and complicates the identification of discrete, therapeutically tractable nodes [121]. Overcoming this challenge will require systems‐level strategies that combine multilayer network reconstruction—integrating phosphoproteomics, ubiquitinomics, histone PTMs, metabolomics, and chromatin accessibility—with state‐stratified PTM profiling to reduce cellular heterogeneity and improve inference accuracy. Complementary CRISPR‐based perturbation libraries targeting PTM modifying enzymes in defined CD8^+^ T cell states can help delineate functional dependencies within these networks [122]. In parallel, time‐resolved PTM mapping following activation or TME mimicry will be essential for distinguishing early initiating modifications from downstream adaptive changes, thereby enabling more precise dissection of causal regulatory events [123, 124].

A second major challenge lies in the technical constraints that limit high‐resolution, spatiotemporal profiling of PTM landscapes in physiologically relevant CD8^+^ T cell populations. Most current PTM datasets are derived from ex vivo–expanded T cells, which fail to recapitulate the metabolic pressure, spatial heterogeneity, and dysfunction gradients present in tumors. Conventional mass spectrometry lacks the sensitivity to detect low‐stoichiometry, transient, or combinatorial modifications, and spatial proteomic platforms rarely resolve PTM states within distinct TME niches. These methodological gaps impede our ability to define how PTMs evolve across activation trajectories or microenvironmental gradients. Addressing this obstacle will require the development of single‐cell–compatible PTM workflows and microfluidic enrichment technologies capable of capturing rare intratumoral T cells with minimal loss of modification integrity. In parallel, adapting advanced spatial imaging modalities—such as imaging mass cytometry, MIBI, MALDI‐MSI, or proximity labeling—to PTM detection will be critical for mapping modification states in situ. Integrating these platforms with multi‐omic inference frameworks and in vivo isotope tracing of metabolic donors will further enable reconstruction of PTM flux across space and time, thereby providing a more faithful representation of PTM dynamics within intact tumors [125, 126].

A further challenge emerges when attempting to therapeutically manipulate PTMs, as these modifications participate in highly interconnected regulatory axes. Perturbing one PTM class often propagates across linked pathways—for example, glycosylation simultaneously influences TCR complex integrity and immune‐checkpoint turnover, while modulation of lactylation alters substrate availability for acetylation, and SUMOylation broadly impacts nuclear trafficking of transcription factors [127, 128, 129]. These pleiotropic consequences narrow therapeutic windows and increase the risk of systemic toxicity or compensatory rewiring. Effective strategies to mitigate this complexity rely on improving the precision and context selectivity of PTM modulation. TME‐responsive prodrugs that activate under hypoxic, acidic, or ROS‐rich conditions can restrict PTM modulation to tumor sites. Engineering PTM enzymes toward altered substrate specificity—rather than global inhibition—offers another promising avenue, exemplified by lenalidomide's ability to reprogram the CRL4^Crbn^ ubiquitin ligase to restore IL‐2 signaling and PD‐1 blockade responsiveness in CD28‐impaired T cells [130]. Additional specificity can be gained through T cell–targeted nanoparticles, bispecific carriers, or engineered T cells designed to express PTM‐resistant signaling modules [131]. Computational modeling of downstream pathway propagation may further guide rational therapeutic design, enabling anticipation of compensatory effects before clinical intervention [132].

The suppressive TME imposes profound and spatially heterogeneous reprogramming of PTM circuits, limiting the efficacy and uniformity of immunotherapy responses. Hypoxia disrupts hydroxylation networks and stabilizes HIF‐driven transcriptional modules; lactate accumulation accelerates histone and non‐histone lactylation, enforcing dysfunction‐associated chromatin states; and nutrient scarcity depletes acetyl‐CoA and SAM, constraining acetylation and methylation required for effector functionality. These alterations vary across hypoxic cores, lactate‐rich zones, and immunosuppressive tumor margins, generating microregional divergence in CD8^+^ T cell differentiation and responsiveness. Overcoming this barrier will depend on constructing spatially resolved PTM atlases capable of identifying niche‐specific vulnerabilities, followed by the development of therapeutics tailored to distinct metabolic microenvironments. Lactate‐buffering strategies, MCT inhibitors, or metabolic alkalinization may be beneficial in acidic niches, whereas HIF‐axis modulation may be more effective in hypoxic regions. Combining PTM modulation with checkpoint inhibitors, co‐stimulatory agonists, or mitochondrial support can counteract spatial heterogeneity and enhance global therapeutic responsiveness. Engineered T cells incorporating PTM‐insulated transcription factors or metabolic stabilizers may further maintain effector identity across hostile tumor niches [121, 133].

Despite these challenges, PTMs remain one of the most promising avenues for next‐generation immunotherapy, offering unmatched precision for tuning CD8^+^ T cell functionality. Their reversibility, context sensitivity, and regulatory centrality make them ideal targets for fine‐scale reprogramming. Realizing this potential will require the construction of comprehensive PTM fate maps that chart modification dynamics from naïve to exhausted states, integrating single‐cell PTMomics, spatial proteomics, and functional multi‐omics to reveal stage‐specific regulatory nodes. Advances in genome engineering enable precise manipulation of PTM sites using CRISPR‐HDR, base editing, or prime editing to create PTM‐mimetic or PTM‐null variants, while synthetic biology offers opportunities to incorporate PTM‐responsive circuits or protective epigenetic modules into engineered T cells. Incorporating PTM modulation into CAR‐T/TCR‐T engineering, checkpoint blockade, metabolic interventions, and targeted protein degradation frameworks may yield therapies with greater durability and breadth across heterogeneous tumors. In parallel, PTM signatures themselves may serve as biomarkers to stratify patients and guide personalized therapeutic combinations, ultimately enabling a new generation of precision immunotherapies grounded in PTM biology.

## 5 Conclusions

CD8^+^ T cells rely on an intricate network of PTMs to sustain their cytotoxic potential, adapt to metabolic fluctuations, and mount durable antitumor responses. These modifications act as molecular integrators that couple antigenic stimulation, nutrient availability, and inflammatory cues to the transcriptional and chromatin programs governing activation, effector acquisition, and memory stability. Within tumors, however, persistent hypoxia, lactate accumulation, and nutrient deprivation reshape these PTM landscapes, stabilizing exhaustion‐associated circuits and constraining the capacity of CD8^+^ T cells to maintain metabolic fitness and cytotoxicity.

A deeper mechanistic understanding of how specific PTM axes orchestrate CD8^+^ T cell fate—both in steady state and under tumor‐derived stress—will be essential for interpreting why some T cells persist and exert control while others become irreversibly dysfunctional. Emerging advances in high‐resolution proteomics, metabolic tracing, and spatial multi‐omics now offer the tools to dissect these regulatory layers with unprecedented precision. Therapeutic strategies that recalibrate PTM‐dependent pathways, reinforce effector programs or buffer T cells against TME‐driven remodeling hold promise for restoring cytotoxic activity in hostile tumor ecosystems. As PTM signatures begin to inform patient stratification and treatment response, integrating PTM biology into immunotherapy design may ultimately enable more durable and predictable reinvigoration of CD8^+^ T cell–mediated tumor control.

## Author Contributions

L.C. and X.Z. contributed to conceptualization, investigation, supervision, writing – review and editing, project administration, and funding acquisition. Z.Z. contributed to investigation, visualization, writing – original draft, and writing – review and editing. X.C. contributed to the investigation, writing – original draft, and writing – review and editing. N.L., M.Z., and W.F. contributed to visualization, writing – original draft, and writing – review and editing. Y.L. and P.L. contributed to writing – original draft and writing – review and editing.

## Funding

This work was supported by the National Natural Science Foundation of China (82372905, 82573074), the Guangdong Provincial Science and Technology Project Foundation (2022A0505050038), the Young Top‐notch Talent of Pearl River Talent Plan (0920220228), and the Science and Technology Program of Guangzhou (No.2025A04J3464).

## Conflicts of Interest

The authors declare no conflicts of interest.

## Data Availability

Data sharing not applicable to this article as no datasets were generated or analysed during the current study.
